# Adaptive genetic diversity and evidence of population genetic structure in the endangered Sierra Madre Sparrow (*Xenospiza baileyi*)

**DOI:** 10.1371/journal.pone.0232282

**Published:** 2020-04-30

**Authors:** José G. Ham-Dueñas, Ricardo Canales-del-Castillo, Gary Voelker, Irene Ruvalcaba-Ortega, Carlos E. Aguirre-Calderón, José I. González-Rojas

**Affiliations:** 1 Laboratorio de Biología de la Conservación y Desarrollo Sustentable. Cd. Universitaria, Universidad Autónoma de Nuevo León, Facultad de Ciencias Biológicas, San Nicolás de los Garza, Nuevo León, México; 2 Department of Wildlife and Fisheries Sciences, Biodiversity Research and Teaching Collections, Texas A&M University, College Station, Texas, United States of America; 3 Instituto Tecnológico de El Salto, El Salto, P.N., Durango, Mexico; University of Iceland, ICELAND

## Abstract

The magnitude and distribution of genetic diversity through space and time can provide useful information relating to evolutionary potential and conservation status in threatened species. In assessing genetic diversity in species that are of conservation concern, several studies have focused on the use of Toll-like receptors (TLRs). TLRs are innate immune genes related to pathogen resistance, and polymorphisms may reflect not only levels of functional diversity, but may also be used to assess genetic diversity within and among populations. Here, we combined four potentially adaptive markers (TLRs) with one mitochondrial (COI) marker to evaluate genetic variation in the endangered Sierra Madre Sparrow (*Xenospiza baileyi*). This species offers an ideal model to investigate population and evolutionary genetic processes that may be occurring in a habitat restricted endangered species with disjunct populations (Mexico City and Durango), the census sizes of which differ by an order of magnitude. TLRs diversity in the Sierra Madre Sparrow was relatively high, which was not expected given its two small, geographically isolated populations. Genetic diversity was different (but not significantly so) between the two populations, with less diversity seen in the smaller Durango population. Population genetic structure between populations was due to isolation and different selective forces acting on different TLRs; population structure was also evident in COI. Reduction of genetic diversity in COI was observed over 20 years in the Durango population, a result likely caused by habitat loss, a factor which may be the main cause of diversity decline generally. Our results provide information related to the ways in which adaptive variation can be altered by demographic changes due to human-mediated habitat alterations. Furthermore, our findings may help to guide conservation schemes for both populations and their restricted habitat.

## Introduction

Given the current biodiversity and environmental crisis, the evaluation of the genetic diversity of endangered species has become a necessary parameter in understanding their population status, resilience, and viability [1–4]. Many studies involving a conservation genetics framework have relied on neutral markers to assess genetic diversity. While neutral markers have been proven useful, potentially adaptive genes may provide insights into causative effects that are directly impacting fitness in animal populations [2,5,6]. Putative adaptive genes, in a conservation genetics context, are markers that may detect patterns of local adaptation due to environmental drivers which may lead to divergent selection among populations. Such divergence may have fitness consequences and as such detecting these differences may contribute to improved conservation strategies, particularly in a regional or global environmental change context [2,5,7].

Toll-like receptors (TLRs) are potentially adaptive genes involved in the innate immune system, acting as components of the first line of defense, by recognizing conserved structural patterns for specific microbial molecules [8]. These receptors are type I transmembrane glycoproteins that play an important role recognizing “pathogen-associated molecular patterns” derived from protozoan, viruses, bacteria and fungi occurrences [9,10]. TLRs evolve rapidly and become highly informative markers owing to the selection pressure from pathogen-host coevolution [10]. From a macro-evolutionary perspective, purifying selection has been observed as the dominant evolutionary force maintaining functional structure in TLRs generally, although episodic positive selection has been also detected in vertebrates [11,12]. In particular, balancing selection on TLRs has been exhibited in birds [10] and mammals [13,14], and such selection preserves high genetic diversity by maintaining adaptive potential. Some studies have identified specific polymorphic sites (i.e. SNPs) that are under selective pressure, and related to susceptibility to specific diseases [14,15].

Genetic drift is the primary mechanism driving loss of evolutionary potential in small populations, and changes in allele frequencies may lead to maladaptive consequences, such as reductions of genetic diversity and fixation of deleterious alleles [16]; nevertheless, natural selection can also be involved in maintaining immune genetic diversity in such populations. Natural selection has been shown to impact immune diversity in both MHC genes [17] and TLRs [18] and indeed, findings suggest that pathogen-mediated selection plays an important role in maintaining this diversity for these genes [14,15]. As such, Toll-like genes have become important molecular markers for addressing questions about how potential functional genetic diversity is involved in micro-evolutionary processes, particularly in species of conservation concern (e.g., threatened or habitat-restricted) [9,19]. Indeed, there is a relationship between survival and TLRs diversity [20–22], but such a relationship is difficult to detect with neutral markers [23] or genome-wide heterozygosity measures [20]. This then suggests that TLRs are a suitable tool for monitoring potentially inbred populations or those with very low population sizes, and indeed, Toll-like gene diversity in threatened species has been assessed in a number of vertebrate taxa [18,20,21,23–29]. The collective results of these studies have offered assessments of population viability, and the genetic consequences of fragmented populations due to habitat loss. However, most TLRs studies of avian taxa were carried out on islands [22,23,26,30–32], as opposed to mainland systems [20,21,25], where some of the former have found that genetic drift outweighs selection [22,30,33]. Because island systems are often more spatially restricted and homogenous in terms of environmental conditions, genetic responses reflected in TLRs may differ among mainland organisms due to exposure to more heterogeneous environments (e.g., more varied geographic and ecological barriers) and, consequently, a potentially higher diversity and abundance of pathogens [26,34,35]. Additionally, while genetic differentiation in TLRs has been detected between avian insular and mainland populations [30,33], population structure among disjunct mainland populations, which may result from demographic trajectories or contemporary human habitat fragmentation, have not been explored.

**Fig 1:**
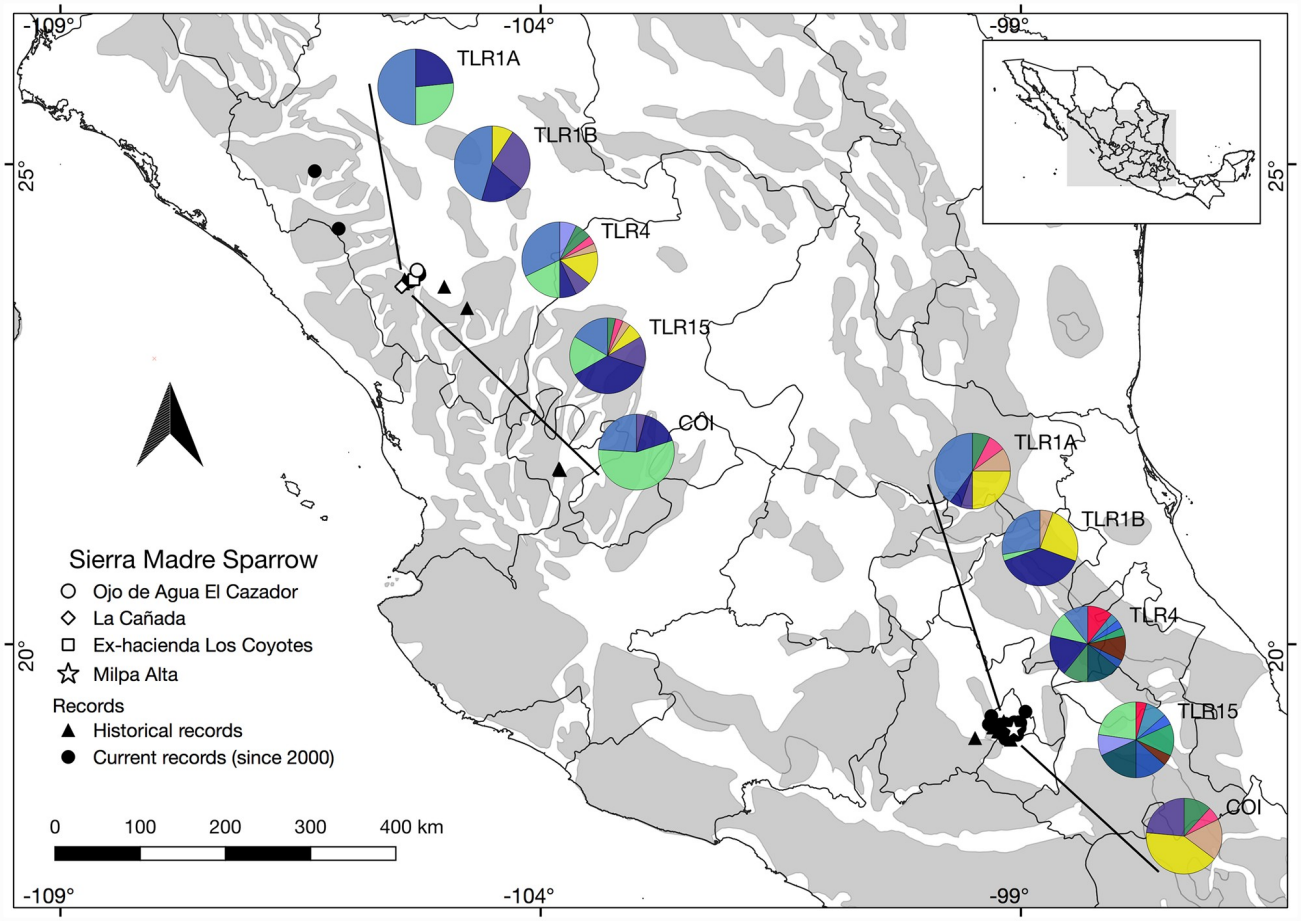
Map of contemporary and historical records of Sierra Madre Sparrow and haplotypes of TLRs. Sampling sites for this study are represented with white symbols, including “Milpa Alta” (star), in Mexico City, and “Ojo de Agua El Cazador” (circle), “La Cañada” (diamond), and “Coyotes” (square) in Durango state. Black shapes indicate historical (triangle) and contemporary records (circles), where georeferenced samples and observations were acquired from Global Biodiversity Information Facility (GBIF) [50]. Haplotype diversity, based on each TLR gene used in this study, are shown in pie charts for both Mexico City and Durango populations.

The Sierra Madre Sparrow (*Xenospiza baileyi*; Passerellidae) [36] is an ideal candidate in which to assess genetic diversity in TLRs. It is an endangered and endemic resident of Mexico that is distributed in two disjunct populations separated by 800 km: one in Durango where it persists in several localities in the Sierra Madre Occidental (SMOc) (C. Aguirre-Calderón *comm*. *pers*.), and one in Mexico City and Morelos where it occurs in the Trans-Mexican Volcanic Belt (TVB) [37,38]. Ecological niche modeling [39] reported no significant ecological dissimilarity between the northern versus southern population localities. However, subtle differences in niche breadth values were detected, particularly in three environmental variables (i.e. precipitation, temperature, and elevation), where the larger southern population was more restricted ecologically. Further, this species is a habitat-restricted, inhabiting patches of subalpine bunch grasslands (*Muhlenbergia spp*., *Festuca spp*., *Calamagrostis tolucensis*., and *Stipa ichu*) isolated among pine forests [38,40,41]. Sierra Madre Sparrows were historically distributed more widely, and in a patchy form, in both the SMOc and TVB, but no evidence exists for a historic connection between these areas. [39,42–44]. Moreover, the Sierra Madre Sparrow has suffered a dramatic habitat reduction owing to anthropogenic land-use modifications, including urbanization, agriculture, and livestock grazing [37,38,41,45,46]. This habitat reduction has been more pronounced in the northern population [38], although bunchgrass areas from the southern Valley of Mexico populations have also been affected. Currently, 50% of habitat available for southern populations has been transformed by human activities [41]. These changes have caused documented historical (in Jalisco, Durango, Morelos and Mexico City) and recent (Ojo de Agua El Cazador, Durango) local extinctions (Fig 1). As a measure of the speed at which human activities continue to impact the Sierra Madre Sparrow, the Ojo de Agua El Cazador population was discovered just 15 years ago [38] with a maximum observed number of 18 sparrows [47], and where the last observation of this species was recorded in 2013 (C. Aguirre-Calderón *comm*. *pers*.). (Fig 1). Censuses reflect trends of population size declines with 18–40 in Durango [47,48] and 2,000 individuals in Mexico City [49].

A recent study of the Sierra Madre Sparrow assessed genetic diversity and population structure using neutral markers, [51] among three sites: “Ojo de Agua El Cazador” (Durango), and “La Cima” and “Milpa Alta” (both Mexico City). Results, based on concatenated mitochondrial coding (COI and ATPase 6 and 8) and non-coding sequences (control region) [51] found low but significant differentiation between the Durango and Mexico City sites; however, few samples from Durango were included (n = 4).

We therefore aim to achieve a broader assessment of the genetic diversity occurring in the Sierra Madre Sparrow, using functional genes related to the innate immune system (i.e., Toll-like receptors). Further, we incorporated additional contemporary samples from both the Durango and Mexico City populations, with previously available material. This increased sampling should provide a more accurate comparison between both populations, and allow us to explore potential causes of specific genetic patterns. Additionally, we utilized the mitochondrial region COI gene to not only compare historical and current genetic diversity, but to also contrast genetic patterns with functional variation in Sierra Madre Sparrow populations. We hypothesize that, given the disparity in population sizes, the Durango population maintains low genetic variation in comparison to the Mexico City population. Given that environmental differences [39] promote alleles fixation via selection (i.e. local adaptation), but temporal isolation can similarly promote genetic divergence [52–54], we also hypothesize that the substantial geographic discontinuity, and environmental conditions between Sierra Madre Sparrow’s populations may promote different evolutionary forces acting independently on each.

## Methods

### Study area and sampling

We collected samples from four localities: a) “Ojo de Agua El Cazador” (2012), “Ex-hacienda Coyotes”, and “La Cañada” (2018) in Durango (SMOc population), and b) San Pablo Oztotepec, “Milpa Alta” (2017) in Mexico City (TVB population) (Fig 1, Table 1 in S1 Text). We actively mist-netted Sierra Madre Sparrows during the breeding season and collected either blood by brachial venipuncture (~20 μl) or one tail feather (outermost rectrix) from each individual. We released them un-harmed at the capture location after samples and standard measurements were taken. Fieldwork was conducted using a bird capture permit issued by SEMARNAT (Secretary for Environmental Management and Natural Resources of Mexico) (SGPA/DGVS/08593/12, SGPA/DGVS/12293/13, SGPA/DGVS/03992/17, and SGPA/DGVS/002953/18).

### DNA extraction, amplification and sequencing

We obtained genomic DNA from both types of samples using the DNeasy blood and tissue extraction kit (Qiagen, Inc.), but used a modified protocol for feather samples by adding DDT (Dithiothretiol) during the digestion process. DNA extractions were quantified using a Qubit 2.0 Fluorometer and visualized by 1.5% agarose gel electrophoresis for quality DNA extraction.

We obtained amplicons of TLRs genes by PCR using primers designed by Chávez-Treviño et al. [55]: TLR1A (ac3TLR1LAF- ac3TLR1LAR), TLR1B (ac3TLR1LBF- ac3TLR1LBR), and TLR4 (ac6TLR4F- ac6TLR4R). We chose these loci for their level of positive selection and to avoid gene duplications and pseudogenes that have been exhibited in other TLRs [19]. We found that TLR1A and TLR1B primers were labeled inaccurately by Chávez-Treviño et al. [55], where the locus tag for forward-reverse primers sequences were interchanged. However, this did not affect subsequent amplification and sequencing processes. For TLR15, we used ac2TLR15R (Chávez-Treviño et al. unpublished) and FinchTLR15F [10] primers, due to a higher specificity for our target species. For the mitochondrial COI gene, we used BirdF1 and BirdR2 primers [56].

We adjusted and performed PCR for TLRs with a total volume of 30 μl, which included approximately 15–50 ng of genomic DNA, 1.5–2.5 mM MgSO_4_, 1X High Fidelity Buffer (600 mM Tris-SO_4_ (pH 8.9), 180 mM (NH_4_)_2_SO_4_), 0.2 mM of each dNTP, 100 ng/μl of each primer, and 0.02 U/μl of Platinum Taq DNA Polymerase High Fidelity (Thermofisher scientific). For TLRs, we set thermal profiles as follows: initial denaturation of 94 °C for 3 min, 30 cycles at 94 °C for 40 s, annealing temperature was specific for each marker: TLR1LB, 56.3 °C; TLR1LB, 54 °C; TLR4, 56 °C; and TLR15, 56 °C; extension step at 72 °C for 1.2 min, and final extension at 72 °C for 10 min. For the mitochondrial COI gene, we defined PCR reaction mixes and thermal profile as described by Hebert et al. (2004). We visualized all PCR products by 1.5% agarose gel electrophoresis. Amplicons were purified and sequenced by MacroGen (MacroGen Inc.). GenBank accession numbers for each TLRs and COI sequences are listed in Supporting information (S1 File; accession numbers MT021464-MT021707).

### Genetic analyses

We visualized, edited and assembled raw TLRs sequences in CodonCode v.5.1.5 (CodonCode Corporation). We first identified poor quality genotyping samples and removed them to avoid undesired effects on downstream analyses. Subsequently, we applied the IUPAC ambiguity code at heterozygous sites across sequences, which we posteriorly aligned for each gene using ClustalW, implemented in Mega v.7 [57]. We compared and validated sequences with our local database and BLAST tools as references. We determined each individual haplotype using the PHASE algorithm [58,59] implemented in DnaSP v.5.10.01 [60], where haplotypes from each individual were selected for subsequent analyses if probability values were greater than 0.6, as an uncertainty threshold estimation associated with each phase call [59].

An appropriate sample size for each locus is needed to represent the genetic variation of animal populations, as it increases the likelihood of detecting private alleles. To assess whether an adequate alleles sampling was conducted, we estimated a rarefaction curve for allele richness and number of samples collected using HP-Rare v.1.0 [61] and compared the allelic richness between populations (*t*-test). We calculated number of segregating sites (*S*), number of haplotypes (*H*), haplotype diversity (*Hd*), and nucleotide diversity (π) for TLRs loci and mitochondrial region COI using DNASP v.5.10.01 [60], and observed (*Ho*) and expected (*He*) heterozygosity using GDA v.1.1. We used *t*-tests to assess whether statistically significant differences in haplotype and nucleotide diversity exist between populations. We tested deviations from Hardy-Weinberg equilibrium, we well as linkage disequilibrium with GENEPOP v.4.2 [62].

To visualize genetic relationships and genetic diversity among Sierra Madre Sparrow populations, we constructed haplotype networks for each gene using inferred haplotype data analyzed in Arlequin v.3.5 [63] that were then plotted with a minimum spanning network in PopArt v.1.7 [64]. To estimate population structure within the mitochondrial COI gene, we used pairwise *F*_ST_ [65–67] and analysis of molecular variance (AMOVA) in Arlequin v.3.5 [63], defining Durango and Mexico City as hierarchical groups. We calculated a fixation index *F*_ST_ [65–67] and a genetic differentiation index *D*_EST_ [68] using GenAlEx v.6.503 [69], to quantify genetic differentiation in TLRs loci. This latter index was used to deal with the dependency of the amount of within-population genetic diversity with *F*_ST_ and related statistics, and to also account for allelic differentiation between subpopulations [68,70–72].

To identify genetically differentiated populations, we analyzed TLR loci using STRUCTURE [73], setting 10 independent runs for K values ranging from 1 to 4, each with 100,000 MCMC iterations after a burn-in of 10,000 iterations, under a model of admixture, and using the locprior option and sampling localities as priors. STRUCTURE analysis assumes that markers are not in linkage disequilibrium, as being in disequilibrium may overestimate clustering [74]. Because linkage disequilibrium was detected between two markers (TLR1A and TLR4), and in order to provide additional insights on population genetic structure, we used a discriminant analysis of principal components (DAPC) [75] using the R package “adegenet” [76]. This method transforms genotypes into uncorrelated components using principal components analysis (PCA). Subsequently, a discriminant analysis is performed on components retained in order to maximize the among-population variation and minimize the variation within groups. This method can be applicable where assumptions such as Hardy-Weinberg equilibrium and Linkage Disequilibrium are not met, while avoiding the use of more conventional approaches (e.g. STRUCTURE). We used a cross-validation function to identify the optimal number of principal components to be retained.

Because recombination may cause bias by increasing the number of false sites positively selected [77], we tested sites under recombination in the alignments of each locus using GARD, as implemented on the Datamonkey web server (www.datamonkey.org). We conducted neutrality tests for haplotype frequencies using DNASP v.5.10.01 [60] including Tajima’s *D* [78], Fu and Li’s *F* [79], *D* [80], Fu’s *Fs* [81], and *R*_*2*_ [82] statistics. We tested for sites under selection using the HyPhy package implemented on the DataMonkey web server (www.datamonkey.org). To detect positive or purifying selection and using the synonymous/non-synonymous ratio, we tested the Mixed Effects Model of Evolution [83] for episodic selection, and the Fast Unconstrained Bayesian AppRoximation model (FUBAR; Murrell et al. 2013) for pervasive selection. The FUBAR method has been observed to detect pervasive selection more efficiently in comparison to Fixed effects likelihood model (FEL) [84]. To detect a site under selection, we used a significance level of < 0.1 for MEME, and a posterior probability of > 0.9 for FUBAR.

To predict whether an amino acid replacement affects protein structure and potentially its functional effect, we used the Protein Variation Effect Analyzer (PROVEAN v.1.1 http://provean.jcvi.org/index.php) [85,86]. This analysis assesses non-synonymous sites to identify which, if any, may likely become deleterious. A protein sequence and amino acid variations were used, then a BLAST search was performed to identify homologous sequences (supporting sequences) and, finally, PROVEAN scores were given, and for which we used a cut-off set to -2.5 for high accuracy [85].

To analyze historical demographic events for each population, we created a Bayesian Skyline Plot for our COI data in BEAST v. 1.8 [87], using a HKY+G nucleotide substitution model and an uncorrelated lognormal strict molecular clock with a divergence rate of 2.5% [88]. Each analysis was run for 10 million generations, with parameters and trees logged every 1000 generations. We examined the results in TRACER v1.5 [89] to confirm convergence and effective sampling of all parameters.

Finally, to evaluate whether there was a temporal change in genetic diversity in Sierra Madre Sparrow populations, we estimated haplotype and nucleotide diversity of COI sequences for each population (Durango and Mexico City), from 15–20 years ago (1999 and 2004; Oliveras de Ita et al. [51]), and contemporary samples from Durango (2012 and 2018) and Mexico City (2017), using DnaSP v.5.10.01 [60]. This temporal comparison was performed using only 612 bp to standardize (in length) the number of samples we could include from GenBank.

## Results

### Genetic diversity and structure

We obtained a total of 37 sequences for each TLR loci (TLR1A, TLR1B, TLR4, and TLR15), with lengths varying from 549 (TLR4) to 1161 bp (TLR15) (Table 1). We did not identify any evidence of premature stop codons or disrupted reading frames, which suggests the absence of pseudogenes. However, we found in five individuals (Mexico City = 3; Durango = 2), a deletion of 24 bp in one of the flanking sections of TLR1B, corresponding to the initiation of the reading frame for the gene. This deletion did not affect the reading frame and the same segment in the other TLR1B samples (those without deletions) was incompletely sequenced near the same flanking section; therefore, we deemed this section as unreliable and discarded it in all samples for subsequent analyses.

**Table 1:** Genetic diversity and polymorphisms for TLR loci in sierra madre sparrow from Durango and Mexico City.

	bp	N	*S*	*H*	*Hd* (SD)	π (SD)	Codons	*dS/dN*	*He*	*Ho*	*F*_IS_	HW (*P*-values)
**Durango**												
**TLR1A**	684	15	3	3	0.646 (0.051)	0.00167 (0.00024)	228	1/2	0.645	0.733	-0.141	0.448
**TLR1B**	831	11	5	4	0.710 (0.062)	0.00246 (0.00022)	277	2/3	0.709	0.818	-0.161	0.502
**TLR4**	549	14	7	9	0.852 (0.044)	0.00318 (0.00029)	183	2/5	0.851	0.857	-0.006	0.160
**TLR15**	1161	15	10	8	0.811 (0.048)	0.00397 (0.00016)	387	8/2	0.811	0.667	0.184	0.388
**COI**	678	25	3	4	0.627 (0.078)	0.00109 (0.00019)	-	-	-	-	-	-
**Mexico City**												
**TLR1A**	684	20	6	7	0.771 (0.047)	0.0019 (0.00024)	228	1/5	0.770	0.500	0.357	0.006*
**TLR1B**	831	18	5	5	0.725 (0.036)	0.00171 (0.00017)	277	2/3	0.725	0.444	0.394	0.032*
**TLR4**	549	14	9	11	0.918 (0.023)	0.00460 (0.00037)	183	5/4	0.917	1.000	-0.093	1.000
**TLR15**	1161	11	15	9	0.896 (0.034)	0.00406 (0.00037)	387	10/5	0.896	0.909	-0.015	0.709
**COI**	678	17	4	5	0.772 (0.070)	0.00171 (0.00028)	-	-	-	-	-	-
**Total**												
**TLR1A**	684	35	7	8	0.757 (0.042)	0.0019 (0.00018)	228	2/5	0.756	0.600	0.210	0.061
**TLR1B**	831	29	7	6	0.750 (0.028)	0.00208 (0.00015)	277	2/5	0.749	0.586	0.221	0.091
**TLR4**	549	28	11	16	0.906 (0.019)	0.00394 (0.00028)	183	5/6	0.905	0.929	-0.026	0.870
**TLR15**	1161	26	16	16	0.901 (0.022)	0.00412 (0.00016)	387	11/5	0.901	0.769	0.149	0.264
**COI**	678	42	5	8	0.829 (0.035)	0.00207 (0.00018)	-	-	-	-	-	-

bp, base pair; N, number of individuals; *S*, segregating sites; *H*, number of haplotypes; *Hd*, haplotype diversity; π, nucleotide diversity; SD, standard deviation; *dS*, synonymous nucleotide changes; *dN*, non-synonymous nucleotide changes; *He*, expected heterozygosity; *Ho*, observed heterozygosity; *F*_IS_, inbreeding coefficient; HW, Hardy-Weinberg disequilibrium.

*Significant *P*-values.

TLR1A and TLR1B from the Mexico City population exhibited deviations from the Hardy-Weinberg equilibrium via the presence of homozygote excess (Table 1). Additionally, significant linkage disequilibrium was detected between the TLR1A and TLR4 loci (*X*^*2*^ = 6.65, *P* = 0.03) in both the Durango population and pooled samples (both populations combined). We found no evidence of recombination in TLR loci using the GARD method. Expected heterozygosity was detected as being higher in Mexico City (*Ho*_mean_ = 0.713; *He*_mean_ = 0.827) than in Durango (*Ho*_mean_ = 0.768; *He*_mean_ = 0.754), although values of observed heterozygosity showed the opposite pattern, likely due to *Ho* values of two loci from Mexico City (TLR1A and TLR1B) being lower (Table 1).

**Fig 2:**
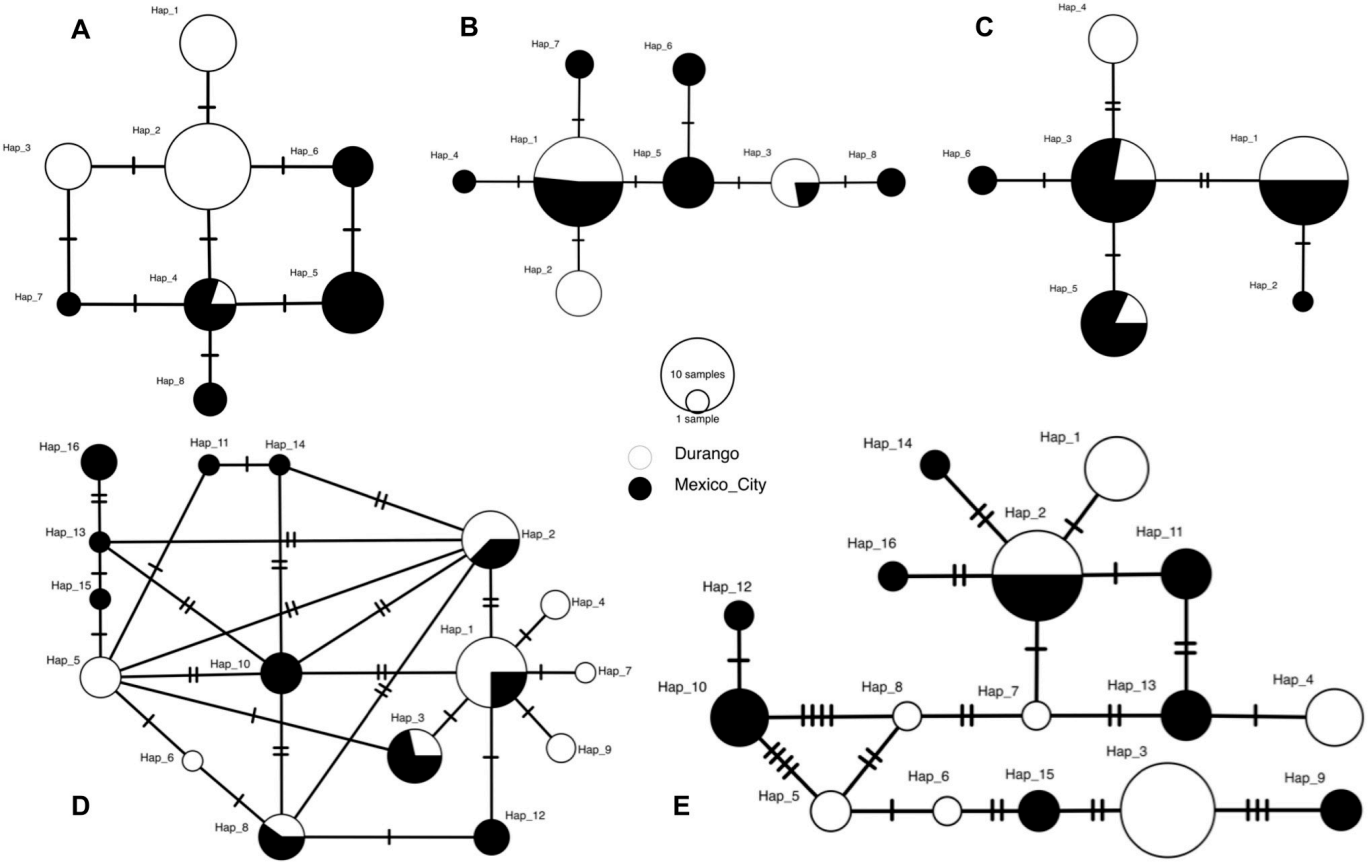
Haplotype networks for mitochondrial and immune-related genes. (A) Mitochondrial region COI; (B) TLR1A; (C) TLR1B; (D), TLR4; and (E) TLR15.

For each population and pooled sample, rarefaction curves of allelic richness reached an asymptote in TLR1A and TLR1B (S1A, S1B and S1C Fig), but this was not evident for TLR4 and TLR15. Mexico City exhibited a higher allelic richness (8.75; SD = 4.5) than Durango (6.25; SD = 2.98); however, the difference was not significant (*t* = -1.02, df = 5.89, *P*-value = 0.34). Across all TLR loci we observed a total of 46 haplotypes in the pooled sample (Mexico City = 32; Durango = 24), where 10 of these were shared haplotypes and 36 of these were unique haplotypes (Mexico City = 22; Durango = 14) (Table 2) (Fig 2). TLR15 showed the highest number of unique haplotypes (15) and had just one shared haplotype between populations. Unique haplotypes in other loci ranged from 3 to 11, while shared haplotypes between populations ranged from 2 to 4 (Fig 2).

**Table 2:** Codon-based analyses of pervasive and episodic selection of TLR loci.

Locus	Selection	Durango		Mexico City		Both	
		MEME	FUBAR	MEME	FUBAR	MEME	FUBAR
**TLR1A**	**+**	0	0	0	0	0	0
	**-**	-	0	-	1 (199)	-	1 (199)
**TLR1B**	**+**	0	0	0	0	0	0
	**-**	-	0	-	0	-	0
**TLR4**	**+**	0	1 (59)	0	2 (59, 144)	0	2 (59, 144)
	**-**	-	1 (23)	-	3 (23, 107, 109)	-	3 (23, 107, 109)
**TLR15**	**+**	0	1 (301)	0	2 (161, 301)	0	1 (301)
	**-**	-	6 (66, 81, 74, 94, 226, 359)	-	7 (66, 81, 94, 111, 164, 226, 272)	-	9 (66, 74, 81, 94, 111, 164, 226, 272, 359)

Codon site that was detected selection is indicated with parenthesis.

All sequences exhibited polymorphic sites with the highest functional variation observed in TLR15, followed by TLR4 (Table 1). The overall nucleotide and haplotype diversity of TLRs was relatively high (*Hd*_mean_ = 0.828, π_mean_ = 0.003) (Table 1). Both estimates of genetic variation were slightly higher in the Mexico City population (*Hd*_mean_ = 0.801, π_mean_ = 0.0027) as compared to Durango (*Hd*_mean_ = 0.729, π_mean_ = 0.0024); however, this difference was not significant (*Hd*: *t* = -1.09, df = 6, *P*-value = 0.31; π: *t* = -0.27, df = 5.22, *P*-value = 0.79).

**Fig 3:**
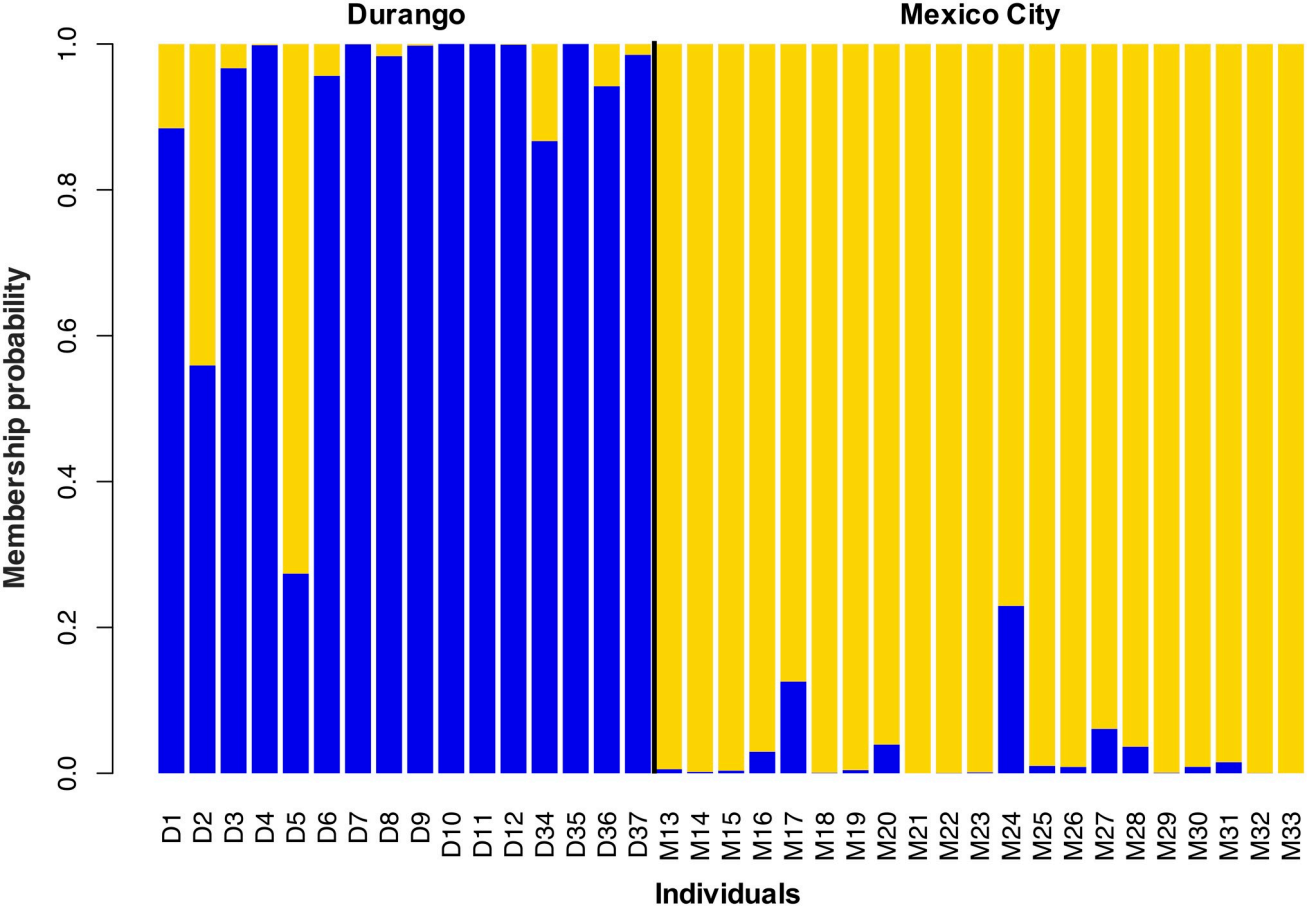
Population structure in Sierra Madre Sparrow from Mexico City and Durango. Plot of membership probabilities from the Discriminant Analysis of Principal Components (DAPC) for TLRs loci, where each column represents an individual.

Based on STRUCTURE analysis, we found evidence of genetic structure in TLR loci between populations. Two runs were performed, one excluding TLR1A and one excluding TLR4, due to our finding of their being in linkage disequilibrium; however, there were no differences in results (S2 Fig). We observed a similar outcome with the discriminant analysis of principal components (DAPC), where each population formed a largely separate cluster that reflected genetic structure (Fig 3). Both the *D*_EST_ and *F*_ST_ statistical analyses, based on allelic differentiation and index fixation respectively, showed significant genetic distinction in all TLR loci (*D*_EST mean_ = 0.344; TLR1A: *D*_EST_ = 0.267, *P* < 0.01; TLR1B: *D*_EST_ = 0.212, *P* < 0.05; TLR4: *D*_EST_ = 0.366, *P* < 0.05; TLR15: *D*_EST_ = 0.736, *P* < 0.01; *F*_ST mean_ = 0.062; TLR1A: *F*_ST_ = 0.068, *P* < 0.01; TLR1B: *F*_ST_ = 0.060, *P* < 0.05; TLR4: *F*_ST_ = 0.041, *P* < 0.05; TLR15: *F*_ST_ = 0.080, *P* < 0.01). Although significant genetic differentiation was observed in TLR4, the magnitude varied according to the test applied.

### Haplotype and site-specific selection

Across TLR loci, we detected 41 SNPs in the Sierra Madre Sparrow data set, of which 20 and 21 SNPs corresponded to synonymous and non-synonymous changes, respectively. Non-synonymous changes were higher for all TLRs, in both populations (Table 1). Regarding site selection analyses, the MEME method did not detect any site under episodic positive selection (Table 2); however, with the FUBAR method we detected sites under positive selection, where two out of three were unique to the Mexico City population (Table 2). We also identified a range of sites as being under negative selection with the FUBAR method; some of these were shared between populations, and some were unique (Table 2). The PROVEAN analysis of non-synonymous sites predicted that four amino acid replacements have a strong effect on protein function (deleterious sites) in three loci (TLR1A, TLR1B, and TLR4), in both populations. We identified two amino acid changes in TLR4 (A7V and S125R) from Durango, and one in TLR1B from the Mexico City population (T147I). One out of four replacements detected with PROVEAN was shared between populations in TLR1A (L74V), with a higher frequency in Mexico City (Table 3).

**Table 3:** PROVEAN analysis with non-synonymous sites of TLR loci in Sierra Madre Sparrow.

Locus	Site (Codon)	Haplotype	AA change	Similar functionality	Allele frequency (Population)	PROVEAN prediction	Score
**TLR1A**	74	3,5,6	Leu/Val	Hydrophobic	4/19 (D/M)	Deleterious	-2.595
	114	7	Met/Ile	Hydrophobic	3 (M)	Neutral	-2.001
	128	8	Arg/Gln	No	3 (M)	Neutral	1.758
	173	6	Asn/Ser	Polar	4 (M)	Neutral	-1.713
	133	3,8	Arg/Lys	Charged	7/5 (D/M)	Neutral	-0.179
**TLR1B**	46	4	Lys/Thr	No	6 (D)	Neutral	-0.345
	102	3,4	Val/Ile	Hydrophobic	9/14 (D/M)	Neutral	0.455
	228	4	Asp/Asn	No	5 (D)	Neutral	-1.419
	147	2	Thr/Ile	No	1 (M)	Deleterious	-5.213
	161	6	Arg/His	No	2 (M)	Neutral	-0.548
**TLR4**	7	4	Ala/Val	Hydrophobic	2 (D)	Deleterious	-2.947
	59	3,5,6,11,15	Asn/His	No	7/7 (D/M)	Neutral	-2.044
	125	9	Ser/Arg	No	2 (D)	Deleterious	-2.574
	139	13,15,16	Gln/His	Polar	5 (M)	Neutral	-1.196
	144	2,5,6,8,10,13,14,15,16	Thr/Ile	No	12/17 (D/M)	Neutral	1.397
	179	2	Gly/Glu	No	5/3 (D/M)	Neutral	1.554
**TLR15**	161	3,5,9,10,14,15	Ala/Asp	No	15/10 (D/M)	Neutral	0.489
	25	10,12	Ser/Asn	Polar	5 (M)	Neutral	0.271
	82	14	Glu/Lys	Charged	1 (M)	Neutral	-1.31
	219	16	Thr/Ile	No	1 (M)	Neutral	-0.488
	301	3,4,9,13,15	Asp/Asn	No	15/7 (D/M)	Neutral	-1.22

AA, amino acid; D = Durango; M = Mexico City. Score < -2.5 indicate a deleterious change.

We also tested whether there were detectable deviations from neutral expectations at the haplotype level. Fu’s *Fs* statistic showed significant negative values in TLR4 for the pooled sample, as well as for each population (Table 4), indicating that purifying selection is the main process operating on this locus. Tajima’s *D*, and both Fu and Li’s *F* and *D* showed departure from neutrality in TLR15 in the Durango population, with a positive value, suggesting that balancing selection (selection that maintains diversity when different alleles are retained within a population) is potentially operating (Table 4). No deviation from neutrality was observed for any loci with the *R*_*2*_ statistic, despite this estimator being a useful test for detecting such deviations in small populations.

**Table 4:** Statistics for departures from neutrality to TLR loci in Sierra Madre Sparrow.

Locus	Tajima´s *D*	Fu & Li´s *D*	Fu & Li´s *F*	Fu´s *Fs*	*R*_*2*_
**Durango**					
**TLR1A**	1.2044	0.9498	1.1847	1.989	0.1908
**TLR1B**	1.4555	1.1756	1.451	2.012	0.2043
**TLR4**	-0.0882	0.5927	0.4538	-2.898*	0.0983
**TLR15**	2.616*	1.4057*	2.0794*	1.64	0.2305
**COI**	-0.17036	-0.20284	-0.2237	-0.477	0.1279
**Mexico City**					
**TLR1A**	-0.2044	1.1919	0.8896	-1.385	0.1085
**TLR1B**	0.4682	0.2182	0.3423	0.533	0.1406
**TLR4**	0.2881	1.3692	1.2164	-3.316*	0.1402
**TLR15**	0.5291	0.4633	0.5621	0.052	0.1513
**COI**	-0.05538	0.23149	0.17668	-0.958	0.1432
**Both**					
**TLR1A**	-0.2624	1.2191	0.8623	-1.499	0.0928
**TLR1B**	0.3748	0.4313	0.4858	0.84	0.1227
**TLR4**	-0.2754	1.4384	1.0209	-7.194*	0.0983
**TLR15**	1.0824	0.2705	0.6495	-1.974	0.1472
**COI**	0.51698	1.11246	1.0861	-1.964	0.1401

*Significant *P*-values.

### Historical genetic diversity

For the mitochondrial COI gene, we obtained a total of 678 bp from 43 sequences, representing samples collected in 2012, 2017, and 2018 (Table 1). Genetic diversity estimates (nucleotide and haplotype diversity) were higher in the Mexico City population as compared to Durango (Table 1). We found a total of eight haplotypes, with only one shared between populations (one individual from Durango and four from Mexico City; Fig 2). This outcome was confirmed with AMOVA, which showed slightly higher variation among populations as compared to within populations (52.31% and 47.69% respectively), and *F*_ST_ values (*F*_ST_ = 0.52, *P*<0.05) that suggested genetic differentiation between populations.

**Fig 4:**
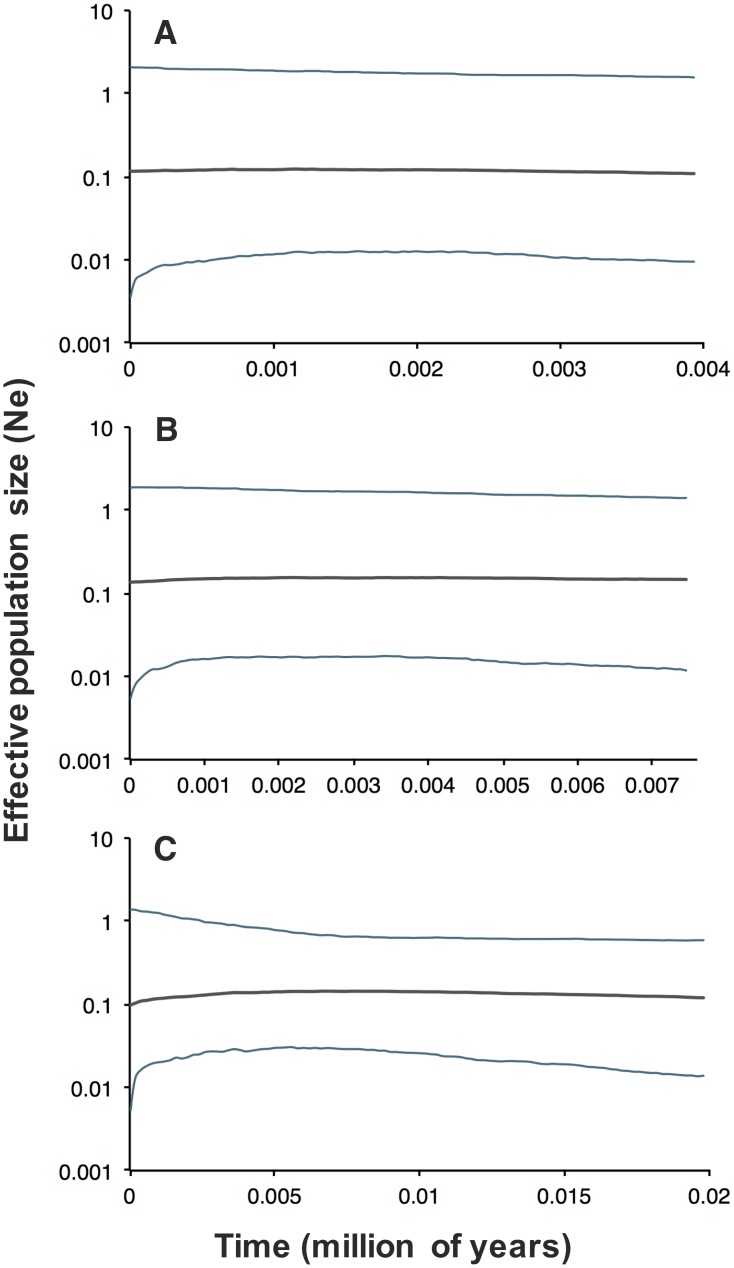
Bayesian skyline plot based on mitochondrial region COI showing change in *N*_*e*_ (effective population size) across time for Sierra Madre populations. Bayesian Skyline Plot analyses were performed for (A) Durango, (B) Mexico City, and (C) both populations. Black solid lines represent median and blue solid lines are the 95% highest posterior density.

**Fig 5:**
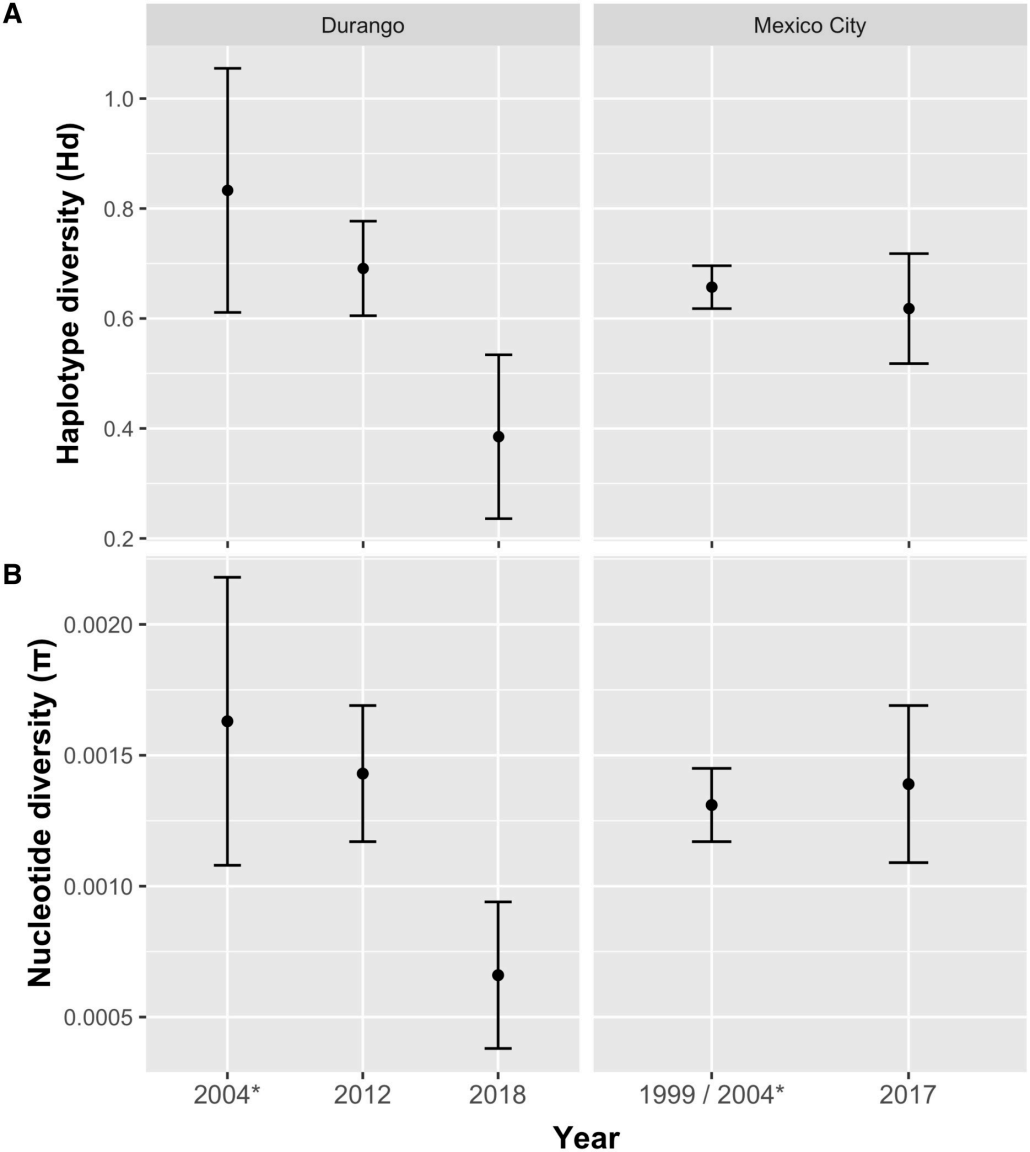
Comparison of genetic diversity variation at different years of sampling. Estimates of (A) haplotype (*Hd*) and (B) nucleotide (π) diversity are represented in each plot. Error bars indicate standard deviation. * Sequences from Olivares de Ita et al. (2012).

We observed no significant values from any neutrality test used for COI (Table 4). We obtained a similar pattern with Bayesian skyline Plots, where a constant effective sample size was detected in both populations through time (Fig 4). In order to determine whether recent temporal changes in genetic variation were evident, we included GenBank samples of COI (612 bp) from individual samples collected in 1999 and 2004 with our COI data. We detected a significant negative trend (*β* = -0.224, 95% CI = -0.306, -0.142) in genetic diversity for the Durango population, where the highest haplotype and nucleotide diversity estimates were from 2004 (*Hd* = 0.833, π = 0.0163), followed by 2012 (*Hd* = 0.691, π = 0.00143) and 2018 (*Hd* = 0.385, π = 0.0066) (Fig 5). We did not detect a temporal change for the Mexico City population (Fig 5).

## Discussion

The Sierra Madre Sparrow offers an ideal model to better understand population and evolutionary genetics processes that occur in endangered species. Its two disjunct populations, each with different demographic history, allows for additional insights into the arrangement of genetic diversity at spatial and temporal scales, and their likely causes. Overall, we observed genetic differentiation between both populations and relatively high genetic diversity across TLRs, which may indicate an independent history for each population.

In general, values for several measures of genetic diversity in TLRs (haplotype and nucleotide diversity) for the Sierra Madre Sparrow (*Hd*_mean_ = 0.828, π_mean_ = 0.003) were similar to or higher than other species with comparably limited range size that are of heightened conservation status [25,26,30,31]. These values were in some cases comparable to species with wide distributions that are of least conservation concern status [26,33], but in general the values are substantially different when compared to species with large population sizes that are categorized as being of least conservation concern [10,30]; such species show a remarkably higher genetic diversity in all TLRs than does the Sierra Madre Sparrow. This interspecific comparison of TLRs variation can be observed in S3 Fig.

Despite the highly restricted distribution and habitat specialization (subalpine bunch grasslands) of the Sierra Madre Sparrow, we observed a large level of neutral diversity (Table 1). We did not detect, however, a signal of expansion or contraction in haplotype networks or Bayesian skyline plots (Fig 4). Furthermore, historical demographic stasis in Sierra Madre Sparrow populations probably maintained its genetic diversity unlike populations of other species, including some North American species with wide distributions, that have undergone demographic expansions from genetic bottlenecks [54,90].

In a demographic context, we found that genetic diversity in Sierra Madre Sparrows was slightly higher in the Mexico City population and, while not significant, this is likely due to differences in effective population size between the two populations. Censuses of contemporary population size vary greatly, where the Durango population is estimated to be between 18–40 individuals [47,48] and the Mexico City population is estimated at around 2,000 individuals (ranged between 1,300–9,999 mature individuals) [49]. This extreme variation among censuses would partially explain some of the variation in genetic diversity we observed, as has been found in several other studies [25,26,30]. However, across the timescale we were able to assess, Bayesian Skyline Plots (Fig 4) suggest that both populations have been stable through time, with similar female effective population sizes (Fig 4). Therefore, we reiterate that the Durango population appears to retain levels of haplotype diversity and nucleotide changes that approach values found in the much larger Mexico City population (Fig 1, Table 1). A similar pattern was found in the Florida Grasshopper Sparrow (*Ammodramus savannarum floridanus*), where, despite a drastic population size reduction over the last 100 years, genetic variation remained similar to other Grasshopper Sparrow populations [91]. While detection of contemporary versus historical demographic changes (e.g. population size variability) may be challenging, we assume that retained ancestral polymorphisms are involved, owing to a large historical effective population size. In fact, some studies argue that estimated genetic variation may depend more on a deeper-time historical changes than on contemporary demographic changes [32,92,93]. This may explain the high genetic diversity in both putative neutral and adaptive loci occurring in the Durango population, despite the census reflecting a very small population size. However, we did found cues of temporal changes in neutral genetic variation, which are discussed below.

Although both Sierra Madre Sparrow populations have somewhat similar levels of TLR haplotype diversity, that diversity is largely partitioned between populations (Figs 2 and 3) as exhibited by the analyses implemented in this study (i.e. Structure, DAPC, *F*_ST_, and *D*_EST_). Through time, this structure could be explained by historical events related to vicariance processes, as has been found in other TLR studies [14,28,30]. Indeed, a similar genetic pattern was observed in lizards that inhabit montane grasslands across Mexico, where Trans-Mexican Volcanic Belt and Sierra Madre Occidental populations were separated genetically [52]; like the Sierra Madre Sparrow, these lizards are also found in bunchgrass. Another possible explanation may be related to more recent events, such as human-driven habitat fragmentation. As there is no documented evidence of geographic connectivity between Sierra Madre Sparrow distributional areas [37,38,43], initial habitat fragmentation into two populations would likely have occurred more than 150 years ago, prior to the oldest recorded specimens (1889). And, considering the rich history of specimen collection in Mexico during the 19th and 20th centuries [94], evidence of connecting populations would probably have been documented [42–44].

In comparison to other bird species with disjunct mainland distributions, the difference in TLRs diversity we found in Sierra Madre Sparrow was higher. However, genetic differentiation in TLRs has been rarely analyzed in mainland bird species [21,25]. One exception is the White-winged flufftail (*Sarothrura ayresi*), where the highly disjunct populations (South Africa and Ethiopia) collectively have between 50–249 mature individuals, exhibit TLRs diversity of just *Hd*_mean_ = 0.0009, and surprisingly show no differentiation between populations [24]. Conversely, genetic differentiation in TLRs was identified between island vs. mainland populations of Song Sparrow (*Melospiza melodia*), indicating a possible isolation effect related to insularity [33], and indeed a similar pattern of differentiation has been observed among isolated oceanic island populations birds [30] and mammals [14,28]. Hence, our findings offer the first avian-based evidence of population genetic structure in TLRs for a mainland system, which in turn suggest signals of local adaptation in each population.

In a natural selection context, neutrality tests (Table 4) found significant positive values in TLR15 for the Durango population, suggesting that balancing selection is occurring. This type of selection has been considered as the main promoter for genetic immune diversity, in both bird [10,95] and mammal species [13,18,96]. Conversely, TLR4 showed significant negative values for Fu’s *Fs* test in both populations, implying purifying selection. While this statistic may be influenced by historic population expansion, no evidence of expansion was detected in the haplotype networks or Bayesian Skyline Plots for our TLRs and COI data, respectively. Moreover, FUBAR analysis indicated that purifying selection was the prevalent process, having been detected in three markers (TLR1A, TLR4, and TLR15; Table 2). Purifying selection would work to both remove deleterious alleles and preserve functionality in TLRs genes [12], and sites under positive selection were detected only in TLR4 and TLR15 (Table 2). This same positive selection dynamic was identified with neutrality tests. A significant deviation from Hardy-Weinberg equilibrium was observed for TLR1A and TLR1B in the Mexico City population due to homozygote excess (Table 1). Potential causes for this deviation may be attributed to selection or inbreeding. In fact, there are studies which assume that selection may be related to heterozygote deficits, especially in TLR1A [25,97]. We detected only one site with purifying selection at this locus, but no site was found to be under selection for TLR1B (Table 2). Finally, inbreeding depression may have impacted these loci, in the Mexico City population; however, no deviation from Hardy-Weinberg equilibrium was detected in the Durango population. This likely suggests a predominant role of selection in the Mexico City population, but detection of which evolutionary forces are acting in each population should be interpreted with caution for potential causes not considered in this study [25]. Overall, our results indicated that evolution was neutral, or almost neutral, in TLR1A and TLR1B, while selection is acting at the codon level in TLR4 and TLR15. TLR4 has been described as a sensor of bacterial lipopolysaccharide [9], while TLR15 is related to the recognition of yeast and bacteria components [98,99]. Both loci are possibly associated with the recognition of protozoan components [23,26,100], while TLR1A and TLR1B have been associated with both protozoans and viruses [97].

At the amino acid level, PROVEAN analyses have predicted deleterious alleles in bird [33] and mammal TLRs [29]. Our analysis indicated that two unique substitutions were detected in Durango in TLR4 and one was detected from Mexico City in TLR1B. Also, a non-synonymous codon substitution in TLR1A (Table 3) revealed a deleterious change, which exhibited remarkably higher frequency in Mexico City. This might be due to linkage disequilibrium with TLR4, where TLR4 maintains a natural selection dynamic. Nevertheless, these genes do not occur on the same chromosome (TLR1A: chromosome 4; TLR4: chromosome 17), from which we may assume a spurious linkage disequilibrium result due to population structure. It might also be related to false-positive amino acid changes due to cut off scores in PROVEAN being set to sensitivity and specificity [86]. It is worth noting that the evidence of non-deleterious alleles, balancing and purifying selection detected in TLR15 suggests a functional constraint, which may prevent harmful mutations of this TLRs that would affect pathogen detection and response [12,101].

The role of ecology may be important in the evolution, maintenance, and diversity of TLRs, with respect to abiotic factors governing the presence and abundance of pathogens. Infectious diseases have been related to climatic factors, mainly precipitation and temperature [102–104], which are clearly heterogeneously distributed in space and time. When tested, no climatic niche differentiation between populations of Sierra Madre Sparrow was found [39]; however, a narrower niche with regard to temperature, precipitation, and elevation was observed for the Mexico City population (temperature = 11.44 °C, SD = 0.54; precipitation = 1293 mm, SD = 29.29; elevation = 3,076 m, SD = 99.2) as compared to Durango (temperature = 13.7 °C, SD = 1.21; precipitation = 735.3 mm, SD = 174.3; elevation = 2,385 m, SD = 246) [39]. We suggest that although the populations occur in similar climatic niches, the exposure to pathogens in each area may be dissimilar. As such, local selective forces might be acting independently to drive variation in TLRs. For example, Coetzer et al. [28] suggested a relationship between rainfall and TLR7 diversity in Vervet monkeys (*Chlorocebus pygerythrus*), which coincided with a strong genetic structuring in TLRs; this pattern was also observed in their mtDNA data [105]. For the Sierra Madre Sparrow, the higher TLR genetic diversity evident in the Mexico City population might be a response to a wider range of pathogen-associated molecules due to higher mean precipitation, which may in turn influence the distribution of diseases such as avian malaria and bacteria [102,103]. Indeed, there is a tantalizing possibility that certain TLRs, such as TLR1A, TLR1B, TLR2B, TLR4, TLR5, and TLR15, bind to proteinaceous ligands and as such might be related to the recognition of Protozoans (e.g., haemosporidians which cause avian malaria) in avian species [23,26,100,106]. As an example of a distinct genetic response, significant temporal changes in TLRs, caused by contemporary bottlenecks, were identified in New Zealand South Island saddleback (*Philesturnus carunculatus*) populations [97]. Specifically, there was an association between a disease-outbreak of haemosporidia parasites and changes in frequencies in two alleles of TLR1A, which suggests an adaptive selection response. Similar responses may explain the reduced genetic variation at this locus, where a specific allele(s) is critical to maintaining responses to a given pathogen (e.g. haemosporidian). This relationship has been observed in other studies as well [26,97].

Finally, genetic drift should not be completely discarded as a potential evolutionary force involved in Sierra Madre Sparrow genetic diversity. For the mitochondrial COI gene, Durango maintained lower genetic diversity than Mexico City as expected from comparative population size, and this lowered diversity may reflect the negative impacts caused by land-use change and habitat fragmentation through time [38,46]. In support of this, we also detected a reduction of COI variation in Durango (Fig 4), over a span of just 20 years. Samples from “Ojo de Agua El Cazador”collected by Oliveras de Ita et al. [38,51] were obtained in 2004, and extirpation has now been reported at this site [96; C. Aguirre-Calderón *comm*. *pers*.]. Similar losses from other historic sites [38], caused by the removal of bunchgrass habitat for crop and livestock activities [40] have almost certainly accelerated this process. In addition, this dramatic reduction of genetic diversity in COI may also be related to the increase in frequency of one or a few haplotypes across the years sampled. Therefore, abrupt population size declines can produce rapid genetic drift locally, and this may be occurring in the Durango population. Finally, no temporal variation was observed in genetic diversity for the Mexico City population, although population size changes are evident from censuses carried out in 1999–2000 (5,380–6,150 adults) [107] and 2016 (1,300 adults) [49].

In conclusion, we have shown that while TLRs diversity is similar (and to a large extent shared) between disjunct Sierra Madre Sparrow populations, local variation may to some extent be driven by differing selective forces, as well as rapid anthropogenic-driven habitat fragmentation. We suggest the need to reappraise the status of Sierra Madre Sparrow and further suggest that due to observed genetic differences (that will certainly increase due to an order of magnitude difference in population size) each population should be considered as an independent unit for conservation (*contra* Oliveras de Ita et al. 2012). “Management unit” (MU) status might be reasonable for each population, because while they are not completely reciprocally monophyletic, significant allele frequency divergence is found in both TLRs and COI [108], and the populations are highly disjunct. Prioritization of conservation areas to maximize evolutionary potential would be another conservation action that can be established; any such action would be enhanced by including other species (e.g. volcano rabbit), which are at risk for local extinctions in the same areas [2]. An assessment of potential disease impacts in both Sierra Madre Sparrow populations is needed to understand the host-pathogen interaction in a meta-population dynamic framework [109], and to assess whether disease is driving genetic variation to some extent. Finally, Toll-like genes provide valuable data with which to evaluate the population status of endangered species, whose adaptive potential may be studied in an environmental global change context.

## Supporting information

S1 Fig Rarefaction curves of TLRs from the sierra madre sparrow samples. Plots of rarefaction curves were obtained from (A) Durango, (B) Mexico City, and (C) both populations.
(TIF)

S2 Fig Structure plots of TLRs from sierra madre sparrow samples. First run was performed with all TLRs except (A) TLR1A and a second run discarding (B) TLR4.
(TIF)

S3 Fig Comparison of nucleotide diversity of TLRs among avian species. Plot show data for nucleotide diversity of TLRs from several species (*Acrocephalus brevipennis*, *A*. *sechellensis*, *A*. *taiti*, *A*. *scirpaceus*, *A*. *arundinaceus* [26], *Anthus berthelotii*, *A*. *campestris* [30], *Petroica australis rakiura* [31], *Melospiza melodia* [33], *Haemorhous mexicanus*, *Falco naumanni* [10], and including Sierra Madre Sparrow populations (D = Durango; MC = Mexico City, and both populations). Each species was categorized as restricted or wide distribution, ordered depending on the contemporary population size (if data is available) according to Birdlife [110]. Also, conservation status is included for each species (LC: Least concern; NT: Near threatened; VU: Vulnerable; EN: Endangered; CR: Critically endangered) [111]. * This specific population inhabits an isolated island, which does not represent the current conservation status, in comparison to the rest of the populations of this bird species.
(TIF)

S1 File Information of the collected specimens and their respective genbank sequences. (DOCX)

S1 Text Genepop file for Toll-like genes obtained from sierra madre sparrow. (TXT)
