# Supplementary figures and images for: Adaptive genetic diversity and evidence of population genetic structure in the endangered Sierra Madre Sparrow (Xenospiza baileyi)
Source: PLoS One. 2020 Apr 30;15(4):e0232282. doi: 10.1371/journal.pone.0232282 (PMC7192469; doi:10.1371/journal.pone.0232282)

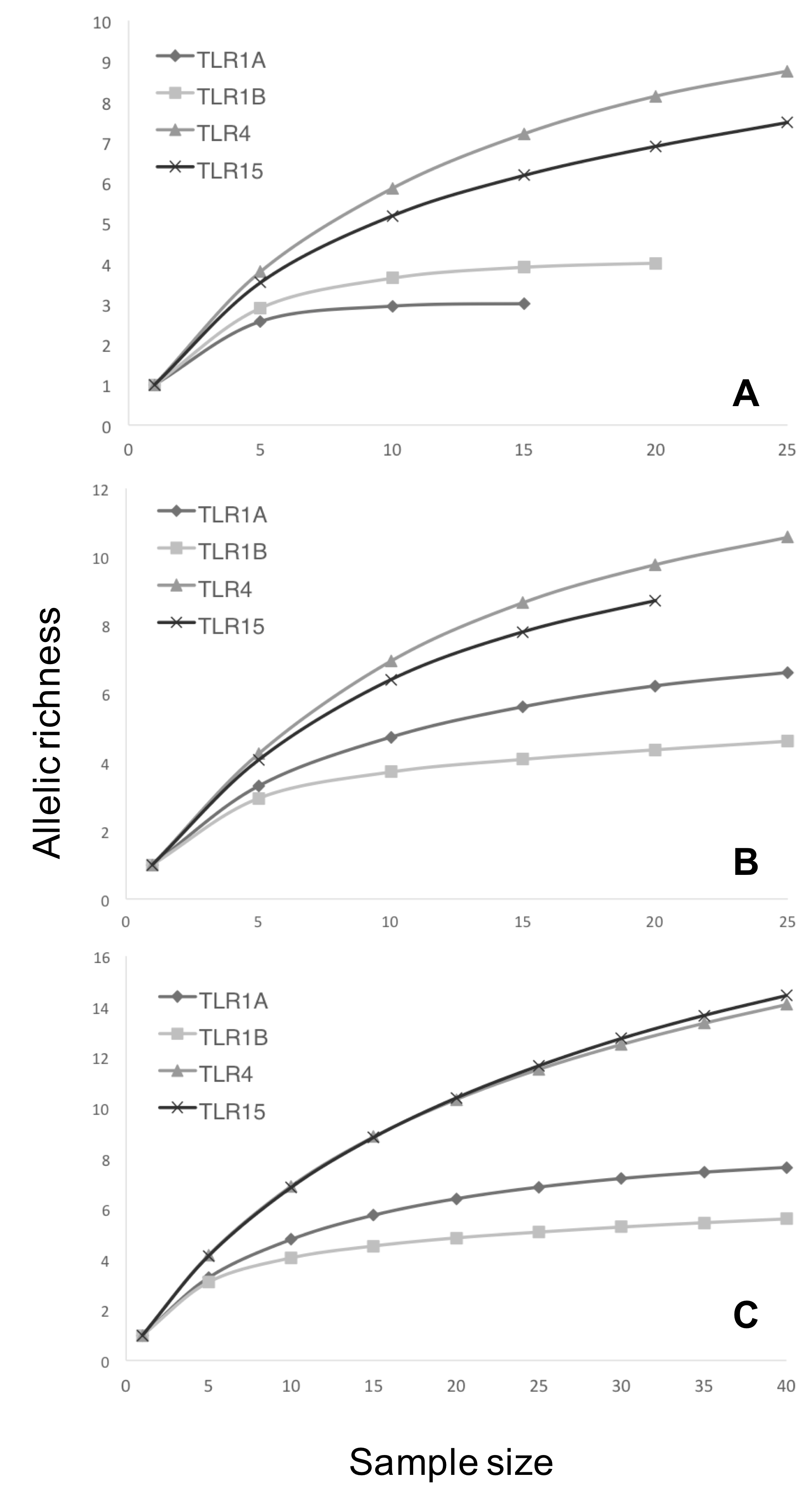

Supplement: S1 Fig — Plots of rarefaction curves were obtained from (A) Durango, (B) Mexico City, and (C) both populations. (TIF) [file pone.0232282.s001.tif]

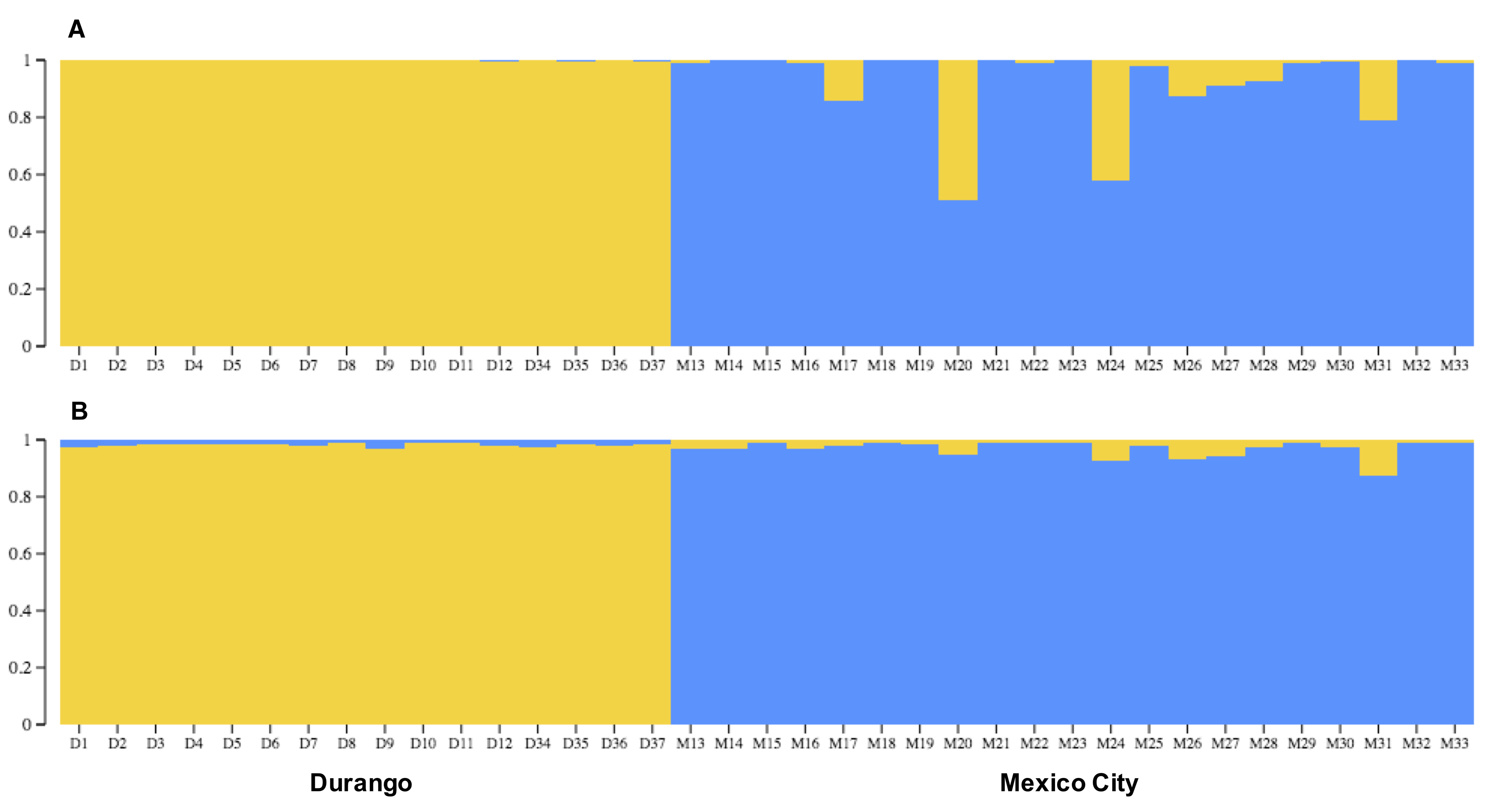

Supplement: S2 Fig — First run was performed with all TLRs except (A) TLR1A and a second run discarding (B) TLR4. (TIF) [file pone.0232282.s002.tif]

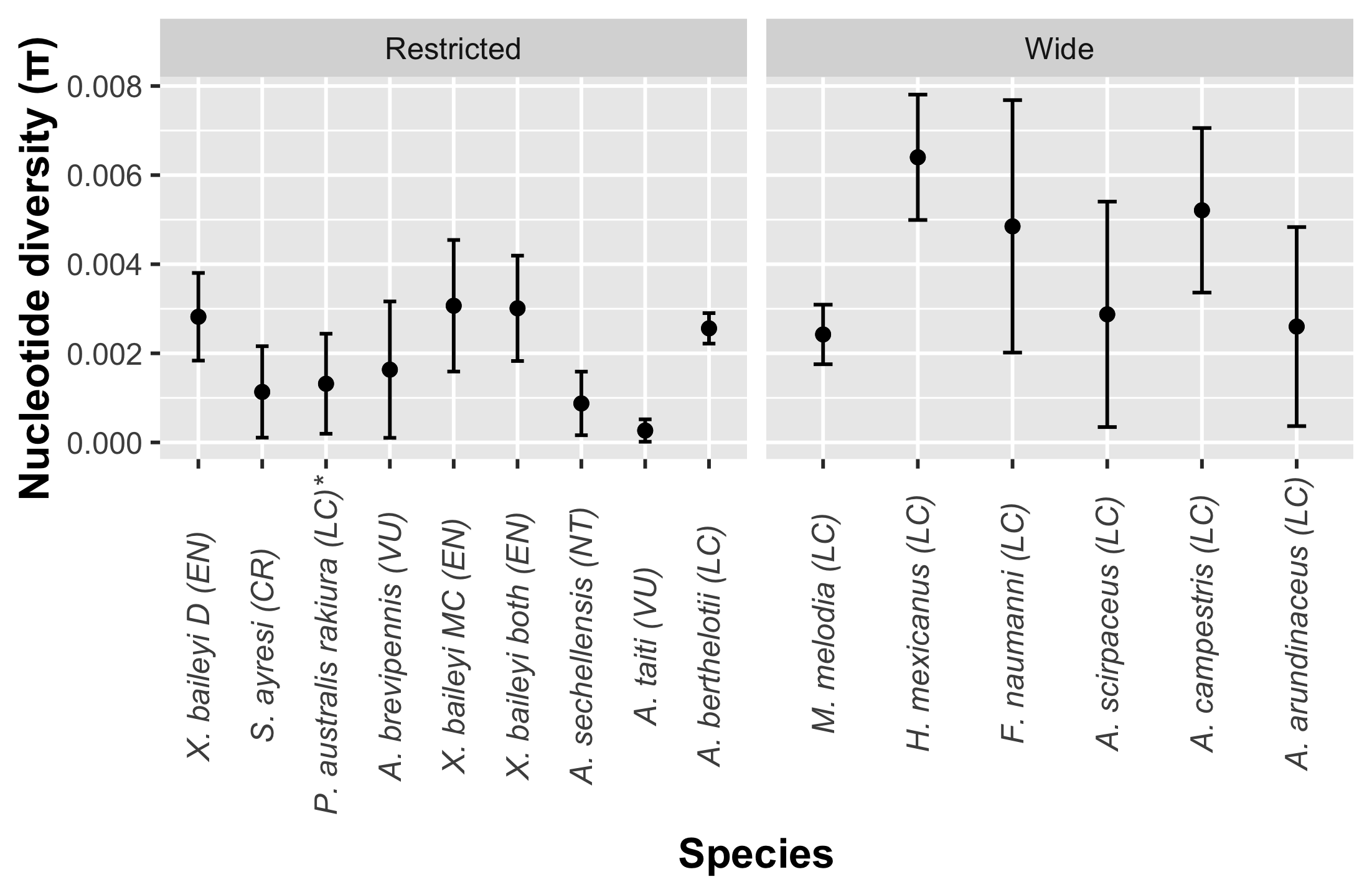

Supplement: S3 Fig — Plot show data for nucleotide diversity of TLRs from several species (Acrocephalus brevipennis, A. sechellensis, A. taiti, A. scirpaceus, A. arundinaceus [26], Anthus berthelotii, A. campestris [30], Petroica australis rakiura [31], Melospiza melodia [33], Haemorhous mexicanus, Falco naumanni [10], and including Sierra Madre Sparrow populations (D = Durango; MC = Mexico City, and both populations). Each species was categorized as restricted or wide distribution, ordered depending on the contemporary population size (if data is available) according to Birdlife [110]. Also, conservation status is included for each species (LC: Least concern; NT: Near threatened; VU: Vulnerable; EN: Endangered; CR: Critically endangered) [111]. * This specific population inhabits an isolated island, which does not represent the current conservation status, in comparison to the rest of the populations of this bird species. (TIF) [file pone.0232282.s003.tif]
