# Supplementary material for: Adaptive genetic diversity and evidence of population genetic structure in the endangered Sierra Madre Sparrow (Xenospiza baileyi)
Source: PLoS One. 2020 Apr 30;15(4):e0232282. doi: 10.1371/journal.pone.0232282 (PMC7192469; doi:10.1371/journal.pone.0232282)
Supplement: S1 File — (DOCX) [file pone.0232282.s004.docx]

**S1 File (APPENDIX)**

Table 1. Information of the collected specimens and the Genbank sequences obtained.

**Toll-like genes**

| **Voucher** | **Isolate** | **Location** | **State** | **Year** | **Latitude** | **Longitude** | **Locus** | **Haplotype** | **Genbank** |
| --- | --- | --- | --- | --- | --- | --- | --- | --- | --- |
| XEN-045 | 1 | Exhacienda Los Coyotes | Durango | 2018 | 23.79823844 | -105.3210778 | TLR1A | 1 | MT021464 |
| XEN-046 | 2 | Exhacienda Los Coyotes | Durango | 2018 | 23.79823844 | -105.3210778 | TLR1A | 1 | MT021465 |
| XEN-047 | 3 | Exhacienda Los Coyotes | Durango | 2018 | 23.79823844 | -105.3210778 | TLR1A | 1 | MT021466 |
| XEN-047 | 3 | Exhacienda Los Coyotes | Durango | 2018 | 23.79823844 | -105.3210778 | TLR1A | 3 | MT021493 |
| XEN-048 | 4 | Exhacienda Los Coyotes | Durango | 2018 | 23.79823844 | -105.3210778 | TLR1A | 1 | MT021467 |
| XEN-049 | 5 | Exhacienda Los Coyotes | Durango | 2018 | 23.79823844 | -105.3210778 | TLR1A | 1 | MT021468 |
| XEN-051 | 7 | Exhacienda Los Coyotes | Durango | 2018 | 23.79823844 | -105.3210778 | TLR1A | 1 | MT021469 |
| XEN-051 | 7 | Exhacienda Los Coyotes | Durango | 2018 | 23.79823844 | -105.3210778 | TLR1A | 2 | MT021485 |
| XEN-052 | 8 | Exhacienda Los Coyotes | Durango | 2018 | 23.79823844 | -105.3210778 | TLR1A | 1 | MT021470 |
| XEN-052 | 8 | Exhacienda Los Coyotes | Durango | 2018 | 23.79823844 | -105.3210778 | TLR1A | 3 | MT021494 |
| XEN-053 | 9 | Exhacienda Los Coyotes | Durango | 2018 | 23.79823844 | -105.3210778 | TLR1A | 1 | MT021471 |
| XEN-053 | 9 | Exhacienda Los Coyotes | Durango | 2018 | 23.79823844 | -105.3210778 | TLR1A | 2 | MT021486 |
| XEN-054 | 10 | Exhacienda Los Coyotes | Durango | 2018 | 23.79823844 | -105.3210778 | TLR1A | 2 | MT021487 |
| XEN-054 | 10 | Exhacienda Los Coyotes | Durango | 2018 | 23.79823844 | -105.3210778 | TLR1A | 3 | MT021495 |
| XEN-058 | 11 | La Cañada | Durango | 2018 | 23.72476037 | -105.4478306 | TLR1A | 1 | MT021472 |
| XEN-058 | 11 | La Cañada | Durango | 2018 | 23.72476037 | -105.4478306 | TLR1A | 2 | MT021488 |
| XEN-060 | 12 | La Cañada | Durango | 2018 | 23.72476037 | -105.4478306 | TLR1A | 2 | MT021489 |
| XEN-060 | 12 | La Cañada | Durango | 2018 | 23.72476037 | -105.4478306 | TLR1A | 3 | MT021496 |
| XEN-001 | 13 | San Pablo Oztotepec | Mexico City | 2017 | 19.11181698 | -99.07532997 | TLR1A | 1 | MT021475 |
| XEN-001 | 13 | San Pablo Oztotepec | Mexico City | 2017 | 19.11181698 | -99.07532997 | TLR1A | 8 | MT021517 |
| XEN-002 | 14 | San Pablo Oztotepec | Mexico City | 2017 | 19.11181698 | -99.07532997 | TLR1A | 1 | MT021476 |
| XEN-003 | 15 | San Pablo Oztotepec | Mexico City | 2017 | 19.11181698 | -99.07532997 | TLR1A | 3 | MT021500 |
| XEN-003 | 15 | San Pablo Oztotepec | Mexico City | 2017 | 19.11181698 | -99.07532997 | TLR1A | 8 | MT021518 |
| XEN-004 | 16 | San Pablo Oztotepec | Mexico City | 2017 | 19.11181698 | -99.07532997 | TLR1A | 1 | MT021477 |
| XEN-004 | 16 | San Pablo Oztotepec | Mexico City | 2017 | 19.11181698 | -99.07532997 | TLR1A | 4 | MT021502 |
| XEN-005 | 17 | San Pablo Oztotepec | Mexico City | 2017 | 19.11181698 | -99.07532997 | TLR1A | 1 | MT021478 |
| XEN-005 | 17 | San Pablo Oztotepec | Mexico City | 2017 | 19.11181698 | -99.07532997 | TLR1A | 5 | MT021504 |
| XEN-006 | 18 | San Pablo Oztotepec | Mexico City | 2017 | 19.11181698 | -99.07532997 | TLR1A | 7 | MT021516 |
| XEN-006 | 18 | San Pablo Oztotepec | Mexico City | 2017 | 19.11181698 | -99.07532997 | TLR1A | 6 | MT021511 |
| XEN-007 | 19 | San Pablo Oztotepec | Mexico City | 2017 | 19.11181698 | -99.07532997 | TLR1A | 5 | MT021505 |
| XEN-009 | 20 | San Pablo Oztotepec | Mexico City | 2017 | 19.11181698 | -99.07532997 | TLR1A | 1 | MT021479 |
| XEN-009 | 20 | San Pablo Oztotepec | Mexico City | 2017 | 19.11181698 | -99.07532997 | TLR1A | 6 | MT021512 |
| XEN-011 | 21 | San Pablo Oztotepec | Mexico City | 2017 | 19.11181698 | -99.07532997 | TLR1A | 7 | MT021515 |
| XEN-012 | 22 | San Pablo Oztotepec | Mexico City | 2017 | 19.11181698 | -99.07532997 | TLR1A | 5 | MT021506 |
| XEN-012 | 22 | San Pablo Oztotepec | Mexico City | 2017 | 19.11181698 | -99.07532997 | TLR1A | 6 | MT021513 |
| XEN-017 | 23 | San Pablo Oztotepec | Mexico City | 2017 | 19.11181698 | -99.07532997 | TLR1A | 5 | MT021507 |
| XEN-018 | 24 | San Pablo Oztotepec | Mexico City | 2017 | 19.11181698 | -99.07532997 | TLR1A | 1 | MT021480 |
| XEN-019 | 25 | San Pablo Oztotepec | Mexico City | 2017 | 19.11181698 | -99.07532997 | TLR1A | 1 | MT021481 |
| XEN-020 | 26 | San Pablo Oztotepec | Mexico City | 2017 | 19.11181698 | -99.07532997 | TLR1A | 1 | MT021482 |
| XEN-032 | 27 | San Pablo Oztotepec | Mexico City | 2017 | 19.11181698 | -99.07532997 | TLR1A | 1 | MT021483 |
| XEN-037 | 29 | San Pablo Oztotepec | Mexico City | 2017 | 19.11181698 | -99.07532997 | TLR1A | 5 | MT021508 |
| XEN-037 | 29 | San Pablo Oztotepec | Mexico City | 2017 | 19.11181698 | -99.07532997 | TLR1A | 8 | MT021519 |
| XEN-039 | 30 | San Pablo Oztotepec | Mexico City | 2017 | 19.11181698 | -99.07532997 | TLR1A | 4 | MT021503 |
| XEN-039 | 30 | San Pablo Oztotepec | Mexico City | 2017 | 19.11181698 | -99.07532997 | TLR1A | 3 | MT021501 |
| XEN-043 | 31 | San Pablo Oztotepec | Mexico City | 2017 | 19.11181698 | -99.07532997 | TLR1A | 1 | MT021484 |
| XEN-113 | 32 | San Pablo Oztotepec | Mexico City | 2017 | 19.11181698 | -99.07532997 | TLR1A | 5 | MT021509 |
| XEN-113 | 32 | San Pablo Oztotepec | Mexico City | 2017 | 19.11181698 | -99.07532997 | TLR1A | 6 | MT021514 |
| XEN-132 | 33 | San Pablo Oztotepec | Mexico City | 2017 | 19.11181698 | -99.07532997 | TLR1A | 5 | MT021510 |
| XEN-055 | 34 | La Cañada | Durango | 2018 | 23.72476037 | -105.4478306 | TLR1A | 1 | MT021473 |
| XEN-055 | 34 | La Cañada | Durango | 2018 | 23.72476037 | -105.4478306 | TLR1A | 3 | MT021497 |
| XEN-056 | 35 | La Cañada | Durango | 2018 | 23.72476037 | -105.4478306 | TLR1A | 2 | MT021490 |
| XEN-056 | 35 | La Cañada | Durango | 2018 | 23.72476037 | -105.4478306 | TLR1A | 3 | MT021498 |
| XEN-057 | 36 | La Cañada | Durango | 2018 | 23.72476037 | -105.4478306 | TLR1A | 2 | MT021491 |
| XEN-057 | 36 | La Cañada | Durango | 2018 | 23.72476037 | -105.4478306 | TLR1A | 3 | MT021499 |
| XEN-059 | 37 | La Cañada | Durango | 2018 | 23.72476037 | -105.4478306 | TLR1A | 1 | MT021474 |
| XEN-059 | 37 | La Cañada | Durango | 2018 | 23.72476037 | -105.4478306 | TLR1A | 2 | MT021492 |
| XEN-045 | 1 | Exhacienda Los Coyotes | Durango | 2018 | 23.79823844 | -105.3210778 | TLR1B | 1 | MT021520 |
| XEN-045 | 1 | Exhacienda Los Coyotes | Durango | 2018 | 23.79823844 | -105.3210778 | TLR1B | 3 | MT021536 |
| XEN-046 | 2 | Exhacienda Los Coyotes | Durango | 2018 | 23.79823844 | -105.3210778 | TLR1B | 1 | MT021521 |
| XEN-046 | 2 | Exhacienda Los Coyotes | Durango | 2018 | 23.79823844 | -105.3210778 | TLR1B | 3 | MT021537 |
| XEN-048 | 4 | Exhacienda Los Coyotes | Durango | 2018 | 23.79823844 | -105.3210778 | TLR1B | 5 | MT021556 |
| XEN-048 | 4 | Exhacienda Los Coyotes | Durango | 2018 | 23.79823844 | -105.3210778 | TLR1B | 4 | MT021550 |
| XEN-049 | 5 | Exhacienda Los Coyotes | Durango | 2018 | 23.79823844 | -105.3210778 | TLR1B | 1 | MT021522 |
| XEN-049 | 5 | Exhacienda Los Coyotes | Durango | 2018 | 23.79823844 | -105.3210778 | TLR1B | 3 | MT021538 |
| XEN-050 | 6 | Exhacienda Los Coyotes | Durango | 2018 | 23.79823844 | -105.3210778 | TLR1B | 1 | MT021523 |
| XEN-051 | 7 | Exhacienda Los Coyotes | Durango | 2018 | 23.79823844 | -105.3210778 | TLR1B | 1 | MT021524 |
| XEN-051 | 7 | Exhacienda Los Coyotes | Durango | 2018 | 23.79823844 | -105.3210778 | TLR1B | 4 | MT021551 |
| XEN-052 | 8 | Exhacienda Los Coyotes | Durango | 2018 | 23.79823844 | -105.3210778 | TLR1B | 1 | MT021525 |
| XEN-053 | 9 | Exhacienda Los Coyotes | Durango | 2018 | 23.79823844 | -105.3210778 | TLR1B | 5 | MT021557 |
| XEN-053 | 9 | Exhacienda Los Coyotes | Durango | 2018 | 23.79823844 | -105.3210778 | TLR1B | 4 | MT021552 |
| XEN-054 | 10 | Exhacienda Los Coyotes | Durango | 2018 | 23.79823844 | -105.3210778 | TLR1B | 3 | MT021539 |
| XEN-054 | 10 | Exhacienda Los Coyotes | Durango | 2018 | 23.79823844 | -105.3210778 | TLR1B | 4 | MT021553 |
| XEN-058 | 11 | La Cañada | Durango | 2018 | 23.72476037 | -105.4478306 | TLR1B | 1 | MT021526 |
| XEN-058 | 11 | La Cañada | Durango | 2018 | 23.72476037 | -105.4478306 | TLR1B | 4 | MT021554 |
| XEN-060 | 12 | La Cañada | Durango | 2018 | 23.72476037 | -105.4478306 | TLR1B | 1 | MT021527 |
| XEN-060 | 12 | La Cañada | Durango | 2018 | 23.72476037 | -105.4478306 | TLR1B | 4 | MT021555 |
| XEN-001 | 13 | San Pablo Oztotepec | Mexico City | 2017 | 19.11181698 | -99.07532997 | TLR1B | 1 | MT021528 |
| XEN-001 | 13 | San Pablo Oztotepec | Mexico City | 2017 | 19.11181698 | -99.07532997 | TLR1B | 3 | MT021540 |
| XEN-002 | 14 | San Pablo Oztotepec | Mexico City | 2017 | 19.11181698 | -99.07532997 | TLR1B | 6 | MT021564 |
| XEN-002 | 14 | San Pablo Oztotepec | Mexico City | 2017 | 19.11181698 | -99.07532997 | TLR1B | 5 | MT021558 |
| XEN-003 | 15 | San Pablo Oztotepec | Mexico City | 2017 | 19.11181698 | -99.07532997 | TLR1B | 5 | MT021559 |
| XEN-005 | 17 | San Pablo Oztotepec | Mexico City | 2017 | 19.11181698 | -99.07532997 | TLR1B | 3 | MT021541 |
| XEN-006 | 18 | San Pablo Oztotepec | Mexico City | 2017 | 19.11181698 | -99.07532997 | TLR1B | 3 | MT021542 |
| XEN-009 | 20 | San Pablo Oztotepec | Mexico City | 2017 | 19.11181698 | -99.07532997 | TLR1B | 1 | MT021529 |
| XEN-011 | 21 | San Pablo Oztotepec | Mexico City | 2017 | 19.11181698 | -99.07532997 | TLR1B | 3 | MT021543 |
| XEN-011 | 21 | San Pablo Oztotepec | Mexico City | 2017 | 19.11181698 | -99.07532997 | TLR1B | 5 | MT021560 |
| XEN-012 | 22 | San Pablo Oztotepec | Mexico City | 2017 | 19.11181698 | -99.07532997 | TLR1B | 3 | MT021544 |
| XEN-017 | 23 | San Pablo Oztotepec | Mexico City | 2017 | 19.11181698 | -99.07532997 | TLR1B | 3 | MT021545 |
| XEN-017 | 23 | San Pablo Oztotepec | Mexico City | 2017 | 19.11181698 | -99.07532997 | TLR1B | 6 | MT021565 |
| XEN-018 | 24 | San Pablo Oztotepec | Mexico City | 2017 | 19.11181698 | -99.07532997 | TLR1B | 1 | MT021530 |
| XEN-018 | 24 | San Pablo Oztotepec | Mexico City | 2017 | 19.11181698 | -99.07532997 | TLR1B | 3 | MT021546 |
| XEN-020 | 26 | San Pablo Oztotepec | Mexico City | 2017 | 19.11181698 | -99.07532997 | TLR1B | 1 | MT021531 |
| XEN-032 | 27 | San Pablo Oztotepec | Mexico City | 2017 | 19.11181698 | -99.07532997 | TLR1B | 1 | MT021532 |
| XEN-032 | 27 | San Pablo Oztotepec | Mexico City | 2017 | 19.11181698 | -99.07532997 | TLR1B | 2 | MT021535 |
| XEN-033 | 28 | San Pablo Oztotepec | Mexico City | 2017 | 19.11181698 | -99.07532997 | TLR1B | 1 | MT021533 |
| XEN-033 | 28 | San Pablo Oztotepec | Mexico City | 2017 | 19.11181698 | -99.07532997 | TLR1B | 3 | MT021547 |
| XEN-037 | 29 | San Pablo Oztotepec | Mexico City | 2017 | 19.11181698 | -99.07532997 | TLR1B | 3 | MT021548 |
| XEN-037 | 29 | San Pablo Oztotepec | Mexico City | 2017 | 19.11181698 | -99.07532997 | TLR1B | 5 | MT021561 |
| XEN-039 | 30 | San Pablo Oztotepec | Mexico City | 2017 | 19.11181698 | -99.07532997 | TLR1B | 5 | MT021562 |
| XEN-043 | 31 | San Pablo Oztotepec | Mexico City | 2017 | 19.11181698 | -99.07532997 | TLR1B | 1 | MT021534 |
| XEN-113 | 32 | San Pablo Oztotepec | Mexico City | 2017 | 19.11181698 | -99.07532997 | TLR1B | 3 | MT021549 |
| XEN-132 | 33 | San Pablo Oztotepec | Mexico City | 2017 | 19.11181698 | -99.07532997 | TLR1B | 5 | MT021563 |
| XEN-045 | 1 | Exhacienda Los Coyotes | Durango | 2018 | 23.79823844 | -105.3210778 | TLR4 | 1 | MT021566 |
| XEN-046 | 2 | Exhacienda Los Coyotes | Durango | 2018 | 23.79823844 | -105.3210778 | TLR4 | 2 | MT021576 |
| XEN-046 | 2 | Exhacienda Los Coyotes | Durango | 2018 | 23.79823844 | -105.3210778 | TLR4 | 3 | MT021584 |
| XEN-048 | 4 | Exhacienda Los Coyotes | Durango | 2018 | 23.79823844 | -105.3210778 | TLR4 | 1 | MT021567 |
| XEN-049 | 5 | Exhacienda Los Coyotes | Durango | 2018 | 23.79823844 | -105.3210778 | TLR4 | 2 | MT021577 |
| XEN-049 | 5 | Exhacienda Los Coyotes | Durango | 2018 | 23.79823844 | -105.3210778 | TLR4 | 4 | MT021591 |
| XEN-050 | 6 | Exhacienda Los Coyotes | Durango | 2018 | 23.79823844 | -105.3210778 | TLR4 | 1 | MT021568 |
| XEN-050 | 6 | Exhacienda Los Coyotes | Durango | 2018 | 23.79823844 | -105.3210778 | TLR4 | 5 | MT021593 |
| XEN-052 | 8 | Exhacienda Los Coyotes | Durango | 2018 | 23.79823844 | -105.3210778 | TLR4 | 1 | MT021569 |
| XEN-052 | 8 | Exhacienda Los Coyotes | Durango | 2018 | 23.79823844 | -105.3210778 | TLR4 | 2 | MT021578 |
| XEN-053 | 9 | Exhacienda Los Coyotes | Durango | 2018 | 23.79823844 | -105.3210778 | TLR4 | 5 | MT021594 |
| XEN-053 | 9 | Exhacienda Los Coyotes | Durango | 2018 | 23.79823844 | -105.3210778 | TLR4 | 6 | MT021597 |
| XEN-054 | 10 | Exhacienda Los Coyotes | Durango | 2018 | 23.79823844 | -105.3210778 | TLR4 | 1 | MT021570 |
| XEN-054 | 10 | Exhacienda Los Coyotes | Durango | 2018 | 23.79823844 | -105.3210778 | TLR4 | 2 | MT021579 |
| XEN-058 | 11 | La Cañada | Durango | 2018 | 23.72476037 | -105.4478306 | TLR4 | 7 | MT021598 |
| XEN-058 | 11 | La Cañada | Durango | 2018 | 23.72476037 | -105.4478306 | TLR4 | 4 | MT021592 |
| XEN-060 | 12 | La Cañada | Durango | 2018 | 23.72476037 | -105.4478306 | TLR4 | 8 | MT021599 |
| XEN-060 | 12 | La Cañada | Durango | 2018 | 23.72476037 | -105.4478306 | TLR4 | 5 | MT021595 |
| XEN-001 | 13 | San Pablo Oztotepec | Mexico City | 2017 | 19.11181698 | -99.07532997 | TLR4 | 8 | MT021601 |
| XEN-001 | 13 | San Pablo Oztotepec | Mexico City | 2017 | 19.11181698 | -99.07532997 | TLR4 | 10 | MT021606 |
| XEN-002 | 14 | San Pablo Oztotepec | Mexico City | 2017 | 19.11181698 | -99.07532997 | TLR4 | 15 | MT021616 |
| XEN-002 | 14 | San Pablo Oztotepec | Mexico City | 2017 | 19.11181698 | -99.07532997 | TLR4 | 16 | MT021617 |
| XEN-003 | 15 | San Pablo Oztotepec | Mexico City | 2017 | 19.11181698 | -99.07532997 | TLR4 | 12 | MT021611 |
| XEN-003 | 15 | San Pablo Oztotepec | Mexico City | 2017 | 19.11181698 | -99.07532997 | TLR4 | 3 | MT021586 |
| XEN-004 | 16 | San Pablo Oztotepec | Mexico City | 2017 | 19.11181698 | -99.07532997 | TLR4 | 10 | MT021607 |
| XEN-004 | 16 | San Pablo Oztotepec | Mexico City | 2017 | 19.11181698 | -99.07532997 | TLR4 | 3 | MT021587 |
| XEN-005 | 17 | San Pablo Oztotepec | Mexico City | 2017 | 19.11181698 | -99.07532997 | TLR4 | 1 | MT021573 |
| XEN-005 | 17 | San Pablo Oztotepec | Mexico City | 2017 | 19.11181698 | -99.07532997 | TLR4 | 3 | MT021588 |
| XEN-006 | 18 | San Pablo Oztotepec | Mexico City | 2017 | 19.11181698 | -99.07532997 | TLR4 | 10 | MT021608 |
| XEN-006 | 18 | San Pablo Oztotepec | Mexico City | 2017 | 19.11181698 | -99.07532997 | TLR4 | 11 | MT021610 |
| XEN-011 | 21 | San Pablo Oztotepec | Mexico City | 2017 | 19.11181698 | -99.07532997 | TLR4 | 10 | MT021609 |
| XEN-011 | 21 | San Pablo Oztotepec | Mexico City | 2017 | 19.11181698 | -99.07532997 | TLR4 | 16 | MT021618 |
| XEN-018 | 24 | San Pablo Oztotepec | Mexico City | 2017 | 19.11181698 | -99.07532997 | TLR4 | 1 | MT021574 |
| XEN-018 | 24 | San Pablo Oztotepec | Mexico City | 2017 | 19.11181698 | -99.07532997 | TLR4 | 2 | MT021581 |
| XEN-019 | 25 | San Pablo Oztotepec | Mexico City | 2017 | 19.11181698 | -99.07532997 | TLR4 | 1 | MT021575 |
| XEN-019 | 25 | San Pablo Oztotepec | Mexico City | 2017 | 19.11181698 | -99.07532997 | TLR4 | 12 | MT021612 |
| XEN-032 | 27 | San Pablo Oztotepec | Mexico City | 2017 | 19.11181698 | -99.07532997 | TLR4 | 2 | MT021582 |
| XEN-032 | 27 | San Pablo Oztotepec | Mexico City | 2017 | 19.11181698 | -99.07532997 | TLR4 | 3 | MT021589 |
| XEN-037 | 29 | San Pablo Oztotepec | Mexico City | 2017 | 19.11181698 | -99.07532997 | TLR4 | 3 | MT021590 |
| XEN-037 | 29 | San Pablo Oztotepec | Mexico City | 2017 | 19.11181698 | -99.07532997 | TLR4 | 16 | MT021619 |
| XEN-039 | 30 | San Pablo Oztotepec | Mexico City | 2017 | 19.11181698 | -99.07532997 | TLR4 | 12 | MT021613 |
| XEN-039 | 30 | San Pablo Oztotepec | Mexico City | 2017 | 19.11181698 | -99.07532997 | TLR4 | 8 | MT021603 |
| XEN-043 | 31 | San Pablo Oztotepec | Mexico City | 2017 | 19.11181698 | -99.07532997 | TLR4 | 2 | MT021583 |
| XEN-043 | 31 | San Pablo Oztotepec | Mexico City | 2017 | 19.11181698 | -99.07532997 | TLR4 | 13 | MT021614 |
| XEN-113 | 32 | San Pablo Oztotepec | Mexico City | 2017 | 19.11181698 | -99.07532997 | TLR4 | 8 | MT021602 |
| XEN-113 | 32 | San Pablo Oztotepec | Mexico City | 2017 | 19.11181698 | -99.07532997 | TLR4 | 14 | MT021615 |
| XEN-055 | 34 | La Cañada | Durango | 2018 | 23.72476037 | -105.4478306 | TLR4 | 1 | MT021571 |
| XEN-055 | 34 | La Cañada | Durango | 2018 | 23.72476037 | -105.4478306 | TLR4 | 2 | MT021580 |
| XEN-056 | 35 | La Cañada | Durango | 2018 | 23.72476037 | -105.4478306 | TLR4 | 1 | MT021572 |
| XEN-056 | 35 | La Cañada | Durango | 2018 | 23.72476037 | -105.4478306 | TLR4 | 9 | MT021604 |
| XEN-057 | 36 | La Cañada | Durango | 2018 | 23.72476037 | -105.4478306 | TLR4 | 8 | MT021600 |
| XEN-057 | 36 | La Cañada | Durango | 2018 | 23.72476037 | -105.4478306 | TLR4 | 5 | MT021596 |
| XEN-059 | 37 | La Cañada | Durango | 2018 | 23.72476037 | -105.4478306 | TLR4 | 9 | MT021605 |
| XEN-059 | 37 | La Cañada | Durango | 2018 | 23.72476037 | -105.4478306 | TLR4 | 3 | MT021585 |
| XEN-045 | 1 | Exhacienda Los Coyotes | Durango | 2018 | 23.79823844 | -105.3210778 | TLR15 | 1 | MT021620 |
| XEN-045 | 1 | Exhacienda Los Coyotes | Durango | 2018 | 23.79823844 | -105.3210778 | TLR15 | 2 | MT021624 |
| XEN-046 | 2 | Exhacienda Los Coyotes | Durango | 2018 | 23.79823844 | -105.3210778 | TLR15 | 1 | MT021621 |
| XEN-047 | 3 | Exhacienda Los Coyotes | Durango | 2018 | 23.79823844 | -105.3210778 | TLR15 | 3 | MT021632 |
| XEN-048 | 4 | Exhacienda Los Coyotes | Durango | 2018 | 23.79823844 | -105.3210778 | TLR15 | 3 | MT021633 |
| XEN-049 | 5 | Exhacienda Los Coyotes | Durango | 2018 | 23.79823844 | -105.3210778 | TLR15 | 3 | MT021634 |
| XEN-049 | 5 | Exhacienda Los Coyotes | Durango | 2018 | 23.79823844 | -105.3210778 | TLR15 | 4 | MT021640 |
| XEN-050 | 6 | Exhacienda Los Coyotes | Durango | 2018 | 23.79823844 | -105.3210778 | TLR15 | 5 | MT021644 |
| XEN-050 | 6 | Exhacienda Los Coyotes | Durango | 2018 | 23.79823844 | -105.3210778 | TLR15 | 3 | MT021635 |
| XEN-051 | 7 | Exhacienda Los Coyotes | Durango | 2018 | 23.79823844 | -105.3210778 | TLR15 | 1 | MT021622 |
| XEN-051 | 7 | Exhacienda Los Coyotes | Durango | 2018 | 23.79823844 | -105.3210778 | TLR15 | 6 | MT021646 |
| XEN-052 | 8 | Exhacienda Los Coyotes | Durango | 2018 | 23.79823844 | -105.3210778 | TLR15 | 3 | MT021636 |
| XEN-052 | 8 | Exhacienda Los Coyotes | Durango | 2018 | 23.79823844 | -105.3210778 | TLR15 | 4 | MT021641 |
| XEN-053 | 9 | Exhacienda Los Coyotes | Durango | 2018 | 23.79823844 | -105.3210778 | TLR15 | 7 | MT021647 |
| XEN-053 | 9 | Exhacienda Los Coyotes | Durango | 2018 | 23.79823844 | -105.3210778 | TLR15 | 3 | MT021637 |
| XEN-054 | 10 | Exhacienda Los Coyotes | Durango | 2018 | 23.79823844 | -105.3210778 | TLR15 | 3 | MT021638 |
| XEN-058 | 11 | La Cañada | Durango | 2018 | 23.72476037 | -105.4478306 | TLR15 | 1 | MT021623 |
| XEN-058 | 11 | La Cañada | Durango | 2018 | 23.72476037 | -105.4478306 | TLR15 | 3 | MT021639 |
| XEN-060 | 12 | La Cañada | Durango | 2018 | 23.72476037 | -105.4478306 | TLR15 | 2 | MT021625 |
| XEN-060 | 12 | La Cañada | Durango | 2018 | 23.72476037 | -105.4478306 | TLR15 | 8 | MT021648 |
| XEN-003 | 15 | San Pablo Oztotepec | Mexico City | 2017 | 19.11181698 | -99.07532997 | TLR15 | 9 | MT021649 |
| XEN-003 | 15 | San Pablo Oztotepec | Mexico City | 2017 | 19.11181698 | -99.07532997 | TLR15 | 10 | MT021651 |
| XEN-006 | 18 | San Pablo Oztotepec | Mexico City | 2017 | 19.11181698 | -99.07532997 | TLR15 | 11 | MT021655 |
| XEN-006 | 18 | San Pablo Oztotepec | Mexico City | 2017 | 19.11181698 | -99.07532997 | TLR15 | 12 | MT021658 |
| XEN-011 | 21 | San Pablo Oztotepec | Mexico City | 2017 | 19.11181698 | -99.07532997 | TLR15 | 11 | MT021656 |
| XEN-011 | 21 | San Pablo Oztotepec | Mexico City | 2017 | 19.11181698 | -99.07532997 | TLR15 | 10 | MT021652 |
| XEN-012 | 22 | San Pablo Oztotepec | Mexico City | 2017 | 19.11181698 | -99.07532997 | TLR15 | 13 | MT021659 |
| XEN-012 | 22 | San Pablo Oztotepec | Mexico City | 2017 | 19.11181698 | -99.07532997 | TLR15 | 10 | MT021654 |
| XEN-017 | 23 | San Pablo Oztotepec | Mexico City | 2017 | 19.11181698 | -99.07532997 | TLR15 | 9 | MT021650 |
| XEN-017 | 23 | San Pablo Oztotepec | Mexico City | 2017 | 19.11181698 | -99.07532997 | TLR15 | 14 | MT021662 |
| XEN-019 | 25 | San Pablo Oztotepec | Mexico City | 2017 | 19.11181698 | -99.07532997 | TLR15 | 13 | MT021660 |
| XEN-019 | 25 | San Pablo Oztotepec | Mexico City | 2017 | 19.11181698 | -99.07532997 | TLR15 | 15 | MT021663 |
| XEN-020 | 26 | San Pablo Oztotepec | Mexico City | 2017 | 19.11181698 | -99.07532997 | TLR15 | 2 | MT021628 |
| XEN-020 | 26 | San Pablo Oztotepec | Mexico City | 2017 | 19.11181698 | -99.07532997 | TLR15 | 15 | MT021664 |
| XEN-033 | 28 | San Pablo Oztotepec | Mexico City | 2017 | 19.11181698 | -99.07532997 | TLR15 | 2 | MT021629 |
| XEN-033 | 28 | San Pablo Oztotepec | Mexico City | 2017 | 19.11181698 | -99.07532997 | TLR15 | 11 | MT021657 |
| XEN-037 | 29 | San Pablo Oztotepec | Mexico City | 2017 | 19.11181698 | -99.07532997 | TLR15 | 2 | MT021630 |
| XEN-037 | 29 | San Pablo Oztotepec | Mexico City | 2017 | 19.11181698 | -99.07532997 | TLR15 | 16 | MT021665 |
| XEN-113 | 32 | San Pablo Oztotepec | Mexico City | 2017 | 19.11181698 | -99.07532997 | TLR15 | 2 | MT021631 |
| XEN-132 | 33 | San Pablo Oztotepec | Mexico City | 2017 | 19.11181698 | -99.07532997 | TLR15 | 13 | MT021661 |
| XEN-132 | 33 | San Pablo Oztotepec | Mexico City | 2017 | 19.11181698 | -99.07532997 | TLR15 | 10 | MT021653 |
| XEN-056 | 35 | La Cañada | Durango | 2018 | 23.72476037 | -105.4478306 | TLR15 | 5 | MT021645 |
| XEN-056 | 35 | La Cañada | Durango | 2018 | 23.72476037 | -105.4478306 | TLR15 | 4 | MT021642 |
| XEN-057 | 36 | La Cañada | Durango | 2018 | 23.72476037 | -105.4478306 | TLR15 | 2 | MT021626 |
| XEN-059 | 37 | La Cañada | Durango | 2018 | 23.72476037 | -105.4478306 | TLR15 | 2 | MT021627 |
| XEN-059 | 37 | La Cañada | Durango | 2018 | 23.72476037 | -105.4478306 | TLR15 | 4 | MT021643 |

**COI gene**

| **Voucher** | **Isolate** | **Location** | **State** | **Year** | **Latitude** | **Longitude** | **Locus** | **Genbank** |
| --- | --- | --- | --- | --- | --- | --- | --- | --- |
| XEN-045 | 1 | Exhacienda Los Coyotes | Durango | 2018 | 23.79823844 | -105.3210778 | COI | MT021677 |
| XEN-047 | 3 | Exhacienda Los Coyotes | Durango | 2018 | 23.79823844 | -105.3210778 | COI | MT021678 |
| XEN-048 | 4 | Exhacienda Los Coyotes | Durango | 2018 | 23.79823844 | -105.3210778 | COI | MT021679 |
| XEN-049 | 5 | Exhacienda Los Coyotes | Durango | 2018 | 23.79823844 | -105.3210778 | COI | MT021680 |
| XEN-050 | 6 | Exhacienda Los Coyotes | Durango | 2018 | 23.79823844 | -105.3210778 | COI | MT021681 |
| XEN-051 | 7 | Exhacienda Los Coyotes | Durango | 2018 | 23.79823844 | -105.3210778 | COI | MT021682 |
| XEN-052 | 8 | Exhacienda Los Coyotes | Durango | 2018 | 23.79823844 | -105.3210778 | COI | MT021683 |
| XEN-053 | 9 | Exhacienda Los Coyotes | Durango | 2018 | 23.79823844 | -105.3210778 | COI | MT021684 |
| XEN-054 | 10 | Exhacienda Los Coyotes | Durango | 2018 | 23.79823844 | -105.3210778 | COI | MT021685 |
| XEN-058 | 11 | La Cañada | Durango | 2018 | 23.72476037 | -105.4478306 | COI | MT021686 |
| XEN-060 | 12 | La Cañada | Durango | 2018 | 23.72476037 | -105.4478306 | COI | MT021687 |
| XEN-001 | 13 | San Pablo Oztotepec | Mexico City | 2017 | 19.11181698 | -99.07532997 | COI | MT021691 |
| XEN-002 | 14 | San Pablo Oztotepec | Mexico City | 2017 | 19.11181698 | -99.07532997 | COI | MT021692 |
| XEN-003 | 15 | San Pablo Oztotepec | Mexico City | 2017 | 19.11181698 | -99.07532997 | COI | MT021693 |
| XEN-004 | 16 | San Pablo Oztotepec | Mexico City | 2017 | 19.11181698 | -99.07532997 | COI | MT021694 |
| XEN-005 | 17 | San Pablo Oztotepec | Mexico City | 2017 | 19.11181698 | -99.07532997 | COI | MT021695 |
| XEN-006 | 18 | San Pablo Oztotepec | Mexico City | 2017 | 19.11181698 | -99.07532997 | COI | MT021696 |
| XEN-009 | 20 | San Pablo Oztotepec | Mexico City | 2017 | 19.11181698 | -99.07532997 | COI | MT021697 |
| XEN-011 | 21 | San Pablo Oztotepec | Mexico City | 2017 | 19.11181698 | -99.07532997 | COI | MT021698 |
| XEN-017 | 23 | San Pablo Oztotepec | Mexico City | 2017 | 19.11181698 | -99.07532997 | COI | MT021699 |
| XEN-018 | 24 | San Pablo Oztotepec | Mexico City | 2017 | 19.11181698 | -99.07532997 | COI | MT021700 |
| XEN-019 | 25 | San Pablo Oztotepec | Mexico City | 2017 | 19.11181698 | -99.07532997 | COI | MT021701 |
| XEN-020 | 26 | San Pablo Oztotepec | Mexico City | 2017 | 19.11181698 | -99.07532997 | COI | MT021702 |
| XEN-032 | 27 | San Pablo Oztotepec | Mexico City | 2017 | 19.11181698 | -99.07532997 | COI | MT021703 |
| XEN-033 | 28 | San Pablo Oztotepec | Mexico City | 2017 | 19.11181698 | -99.07532997 | COI | MT021704 |
| XEN-043 | 31 | San Pablo Oztotepec | Mexico City | 2017 | 19.11181698 | -99.07532997 | COI | MT021705 |
| XEN-113 | 32 | San Pablo Oztotepec | Mexico City | 2017 | 19.11181698 | -99.07532997 | COI | MT021706 |
| XEN-132 | 33 | San Pablo Oztotepec | Mexico City | 2017 | 19.11181698 | -99.07532997 | COI | MT021707 |
| XEN-056 | 35 | La Cañada | Durango | 2018 | 23.72476037 | -105.4478306 | COI | MT021688 |
| XEN-057 | 36 | La Cañada | Durango | 2018 | 23.72476037 | -105.4478306 | COI | MT021689 |
| XEN-059 | 37 | La Cañada | Durango | 2018 | 23.72476037 | -105.4478306 | COI | MT021690 |
| 1201 | 38 | Ojo de Agua El Cazador | Durango | 2012 | 23.895664 | -105.287154 | COI | MT021666 |
| 1202 | 39 | Ojo de Agua El Cazador | Durango | 2012 | 23.895664 | -105.287154 | COI | MT021667 |
| 1204 | 40 | Ojo de Agua El Cazador | Durango | 2012 | 23.895664 | -105.287154 | COI | MT021668 |
| 1205 | 41 | Ojo de Agua El Cazador | Durango | 2012 | 23.895664 | -105.287154 | COI | MT021669 |
| 1206 | 42 | Ojo de Agua El Cazador | Durango | 2012 | 23.895664 | -105.287154 | COI | MT021670 |
| 1207 | 43 | Ojo de Agua El Cazador | Durango | 2012 | 23.895664 | -105.287154 | COI | MT021671 |
| 1208 | 44 | Ojo de Agua El Cazador | Durango | 2012 | 23.895664 | -105.287154 | COI | MT021672 |
| 1209 | 45 | Ojo de Agua El Cazador | Durango | 2012 | 23.895664 | -105.287154 | COI | MT021673 |
| 1210 | 46 | Ojo de Agua El Cazador | Durango | 2012 | 23.895664 | -105.287154 | COI | MT021674 |
| 1211 | 47 | Ojo de Agua El Cazador | Durango | 2012 | 23.895664 | -105.287154 | COI | MT021675 |
| 1212 | 48 | Ojo de Agua El Cazador | Durango | 2012 | 23.895664 | -105.287154 | COI | MT021676 |
